# Integration of circulating microRNAs and transcriptome signatures identifies early‐pregnancy biomarkers of preeclampsia

**DOI:** 10.1002/ctm2.1446

**Published:** 2023-10-31

**Authors:** Hooman Mirzakhani, Diane E. Handy, Zheng Lu, Ben Oppenheimer, Augusto A. Litonjua, Joseph Loscalzo, Scott T. Weiss

**Affiliations:** ^1^ Channing Division of Network Medicine Department of Medicine Harvard Medical School Brigham and Women's Hospital Boston Massachusetts USA; ^2^ Division of Cardiovascular Medicine Department of Medicine Brigham and Women's Hospital Harvard Medical School Boston Massachusetts USA; ^3^ Division of Pediatric Pulmonary Medicine Department of Pediatrics Golisano Children's Hospital at Strong University of Rochester Medical Center Rochester New York USA

**Keywords:** circulating RNA, gene expression, hypertension, microRNA expression, preeclampsia, pregnancy, systems biology, trophoblast

## Abstract

**Background:**

MicroRNAs (miRNAs) have been implicated in the pathobiology of preeclampsia, a common hypertensive disorder of pregnancy. In a nested matched case‐control cohort within the Vitamin D Antenatal Asthma Reduction Trial (VDAART), we previously identified peripheral blood mRNA signatures related to preeclampsia and vitamin D status (≤30 ng/mL) during gestation from 10 to 18 weeks, using differential expression analysis.

**Methods:**

Using quantitative PCR arrays, we conducted profiling of circulating miRNAs at 10–18 weeks of gestation in the same VDAART cohort to identify differentially expressed (DE) miRNAs associated with preeclampsia and vitamin D status. For the validation of the expression of circulating miRNA signatures in the placenta, the HTR‐8/SVneo trophoblast cell line was used. Targets of circulating miRNA signatures in the preeclampsia mRNA signatures were identified by consensus ranking of miRNA‐target prediction scores from four sources. The connected component of target signatures was identified by mapping to the protein‐protein interaction (PPI) network and hub targets were determined. As experimental validation, we examined the gene and protein expression of IGF1R, one of the key hub genes, as a target of the DE miRNA, miR‐182‐5p, in response to a miR‐182‐5p mimic in HTR‐8/SVneo cells.

**Results:**

Pregnant women with preeclampsia had 16 circulating DE miRNAs relative to normal pregnancy controls that were also DE under vitamin D insufficiency (9/16 = 56% upregulated, FDR < .05). Thirteen miRNAs (13/16 = 81.3%) were detected in HTR‐8/SVneo cells. Overall, 16 DE miRNAs had 122 targets, of which 87 were unique. Network analysis demonstrated that the 32 targets of DE miRNA signatures created a connected subnetwork in the preeclampsia module with CXCL8, CXCL10, CD274, MMP9 and IGF1R having the highest connectivity and centrality degree. In an in vitro validation experiment, the introduction of an hsa‐miR‐182‐5p mimic resulted in significant reduction of its target IGF1R gene and protein expression within HTR‐8/SVneo cells.

**Conclusions:**

The integration of the circulating DE miRNA and mRNA signatures associated preeclampsia added additional insights into the subclinical molecular signature of preeclampsia. Our systems and network biology approach revealed several biological pathways, including IGF‐1, that may play a role in the early pathophysiology of preeclampsia. These pathways and signatures also denote potential biomarkers for the early stages of preeclampsia and suggest possible preventive measures.

## Graphical abstract

1. In early pregnancy, we identified 16 differentially expressed circulating miRNAs associated with both preeclampsia and vitamin D status.
2. Integration of miRNA signatures with preeclampsia transcriptomic signatures revealed 32 unique targets of miRNA signatures.
3. The expression of 13 miRNA signatures were validated in HTR‐8/SVneo cell line.
4. Transfection of hsa‐miR‐182‐5p to HTR‐8/SVneo cell downregulated both IGF1R protein and mRNA expressions.

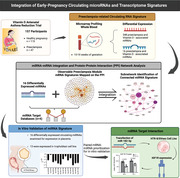

## 1 BACKGROUND

Preeclampsia (PE) occurs in up to 8% of pregnancies.^1^, ^2^ PE, a complex hypertensive disorder that develops during pregnancy, stands as a significant contributor to maternal mortality and perinatal morbidity and mortality. Its effects also extend into long term, impacting on both the mother and the newborn.^3^, ^4^ Early identification of high‐risk pregnancies could prompt more targeted and timely interventions to prevent the onset of PE and to minimise the risks of its complications.^5^ Preeclampsia is believed to arise from inadequate placental development attributed to issues within the blood vessels that provide its supply. However, the precise underlying cause remains incompletely comprehended.^6^ PE is often diagnosed in the later stages of pregnancy (90% of cases are diagnosed after 34 weeks of gestation in the United States), with the only cure being the delivery of the baby and placenta. Identification of a biological marker could not only assist with early detection, but could also help unravel the complex pathobiology of the disease.

Biological networks serves as invaluable platforms for comprehending systems‐level properties and offer a means to contextualise genomic data within the framework of health and disease.^7^, ^8^ Differential gene expression has been helpful as a basis for constructing disease‐specific networks,^9^, ^10^ as genes and their dysregulated expression are among the key driving factors in the development of complex disease.^11^ The identification of active gene modules has played a pivotal role in guiding interventions.^12^ Utilising module‐based biomarkers has demonstrated enhanced predictive power and reproducibility when compared to individual biomarkers. Moreover, active gene modules have the potential to unveil pathway‐centric insights that are supported by multiple lines of evidence through integrative omics analysis. Consequently, these modules can offer mechanistic explanations for complex traits.^12^

In our recent study of circulating mRNA expression profiling,^2^ we discovered a PE‐related gene signature consisting of 348 differentially expressed (DE) genes. These genes were found to be associated with vitamin D status during the gestational period of 10−18 weeks. This association was detected in pregnant women who later developed PE, in contrast to those with uncomplicated pregnancies. Statistically significant findings were observed in both discovery and replication cohorts (Vitamin D Antenatal Asthma Reduction Trial [VDAART, FDR < .05] and the OMEGA cohort [*p* < .05]).^2^ Mapping of this gene set to the physical interactome (protein‐protein interaction network, PPI) suggested that a majority of these gene products comprise a discrete module or subnetwork, including some nodes previously reported in association with PE that demonstrated a high degree of connectivity (direct interaction) with their neighbours in the disease module.^2^ MicroRNAs (miRNAs) exert posttranscriptional control over gene expression and often target the central nodes within an active gene module.^13^ Impairment in the regulation of miRNAs has been implicated in the pathobiology of complex disorders, including PE.^14^, ^15^, ^16^, ^17^ Furthermore, circulating miRNAs as well as extracellular vesicle miRNAs have been proposed as biomarkers for early developmental stages of disease.^18^ More specifically, expression of circulating miRNAs has been shown to differ between pregnant women and nonpregnant women.^19^, ^20^, ^21^ This knowledge suggests that pregnancy‐related miRNAs could be potential markers for adverse pregnancy outcomes.^22^, ^23^

Investigating genomic signatures linked with trophoblast invasion and angiogenesis during the period of rapid placental growth and the subclinical stage of PE (10−20 weeks' gestation) can yield valuable insights into alterations in placental function associated with PE. Given the inherent challenges in accessing placentas during early pregnancy, the exploration of blood‐based markers that could mirror placental activity remains pertinent. Although limited, some studies have examined miRNA expression in peripheral blood during early pregnancy in relation to PE.^24^ Analysing PE‐associated miRNAs and their interaction with mRNA through expression profiles and disease‐specific network analysis holds the potential to shed lights on the intricate pathobiology of PE, offering and novel opportunities for prevention and diagnostics. The potential impact of vitamin D intake on the expression of several circulating miRNAs has been demonstrated in healthy status.^25^ Furthermore, a study has suggested the existence of a potential correlation between miRNAs associated with PE and vitamin D (25OHD) levels in the placenta, which may be reflected in peripheral blood.^26^

Accordingly, the primary aim of this study is to identify circulating miRNAs associated with subclinical PE during early stages of pregnancy (10–18 weeks of gestation) and to discover the target genes that these DE miRNAs regulated within the PE‐related disease module, that is, DE transcriptome signatures during the same gestational period. For this investigation, we used the same VDAART nested case‐control cohort that was used for the prior whole blood gene expression study and construction of the PE‐related disease module. We also explored whether any one of the PE‐associated miRNAs might be associated with vitamin D sufficiency status (25‐hyroxy vitamin D [25OHD] ≥30 ng/mL). Further, we used the HTR‐8/SVneo trophoblast cell line to validate the expression of the circulating miRNA signatures in placenta.

## 2 METHODS

### 2.1 VDAART population

VDAART (www.vdaart.com) was sponsored by the National Heart, Lung, and Blood Institute (NHLBI) and registered at ClinicalTrials.gov (NCT00920621). Ethical approval for VDAART was obtained from the Institutional Review Boards (IRB) at the participating Clinical Centers, which included Washington University in St. Louis, Boston Medical Center, and Kaiser Health Care San Diego. Additionally, approval was obtained from the Data Coordinating Center (DCC) at Brigham Women's Hospital (Mass General Brigham Health Care System, Boston, MA). All pregnant women enrolled in VDAART consented for their participated during the initial enrolment visit. The study population for the VDAART trial comprised pregnant individuals with singleton pregnancies, recruited between the 10th and 18th weeks of gestation, as part of a multicentre, randomised trial. The primary objective of this trial was to evaluate specific pregnancy outcomes. Participants were randomly allocated to one of two groups: the vitamin D (cholecalciferol) group, which received a daily dosage of 4000 IU (equivalent to 100 μg/day), or the placebo group. In addition to this randomisation, all pregnant women were supplied with prenatal vitamins containing 400 IU (10 μg/day) of cholecalciferol. As a result, the vitamin D treatment group received a cumulative daily intake of 4400 IU/day (110 μg/day), while the placebo group received 400 IU/day of vitamin D. The clinical progress of the pregnant women was meticulously tracked from the point of randomisation, which occurred between the 10th and 18th weeks of pregnancy, all the way through to the conclusion of their pregnancies. This comprehensive monitoring aimed to investigate predefined pregnancy outcomes, specifically focusing on conditions such as PE, preterm birth, and term deliveries. The exclusion criteria for enrolment in the VDAART study encompassed several factors. These criteria included refraining from smoking for at least one month prior to enrolment, as well as the use of alternative nicotine products like nicotine patch for a minimum of one month before enrolment. Additionally, individuals with chronic conditions such as diabetes mellitus and ongoing treatment for chronic hypertension, those with multiple gestation pregnancies, pregnancies achieved through assisted reproduction techniques like IUI and IVF, as well as individuals with parathyroid and thyroid disease, kidney stones or sarcoidosis were excluded from the study. Furthermore, participants taking vitamin D supplements exceeding 2000 IU per day or those who had used illicit drugs within the past six months were not eligible for enrolment.^27^, ^28^ The specifies of the trial design and the original clinical trial finding regarding the impact of vitamin D supplementation on asthma and recurrent wheezing in the offspring of the mothers, as well as the association between vitamin D and PE, have been previously published.^2^, ^27^, ^28^

### 2.2 Study participants and their transcriptome data

The participants in this study investigating PE‐associated miRNAs were drawn from a nested case‐control group, consisting of 157 individuals, which was selected from the largest intent‐to‐treat (ITT) VDAART cohort, comprising 816 pregnant participants. Within this ITT cohort, there were 67 cases who developed PE during the course of the study. Among these PE cases, 47 individuals (70.14%) had provided RNA samples at the time of trial enrolment (between 10−18 weeks of gestation) and were included in both the mRNA and the miRNA expression profiling substudy. Controls for this study were chosen from the VDAART participants based on specific matching criteria, including age (within a 5‐year range), race, and the centre where they were enrolled. This matching process ensured that each participant with PE had at least two matched controls, resulting in a total of 110 control participants (N_controls = 110). In a subsequent postmatching comparison, there was no statistically significant difference (*p* values > .05) observed in terms of age, race or maternal gestational age during early pregnancy as well as foetal sex and intervention arms when comparing the group of 47 cases with PE and the 110 women who experienced uncomplicated pregnancies. Table 1 demonstrates the study participants’ characteristics. The results for the mRNA expression study have been published.^2^ The replicated mRNA signature associated with PE in our prior report (*N* = 348) from the aforementioned study cohort was used for integration with profiled miRNAs in this study.^2^

**TABLE 1:** Clinical characteristics of the VDAART participants used for mRNA and miRNA expression profiling and integration analysis.

Characteristics	Genomic (mRNA‐miRNA) study cohort in VDAART	
	Preeclampsia (*n* = 47)	Healthy Pregnancy (*n* = 110)
Gestational age (weeks)		
Range	10‐18	10‐18
Age in years		
Mean (SD)^a^	25.8 (5.0)	26.6 (5.1)
Race (*n* ^b^)		
White	17	42
African American	22	61
Other	8	7
Serum 25OHD^c^ at enrolment (ng/mL)		
Mean (SD)	19.7 (8.3)	24.4 (14.5)
Range (ng/mL)	7–36.5	5.1–80
BMI at enrolment (kg/m^2^)		
Mean (SD)	31.2 (8.0)	27.9 (7.9)
Missing (*n*)	0	7
Foetal sex (*n*)		
Female	20	58
Male	27	52
Intervention arm (*n*)		
Placebo	24	51
Treatment	23	59

^a^Standard deviation.

^b^Number.

^c^25‐hydroxy vitamin D.

### 2.3 Study outcome and 25OHD measurement

The emergence of PE was a prespecified secondary result of the trial and an adverse event.

After delivery, medical records were abstracted for each participant. The diagnosis of PE was validated through a thorough process. A committee comprising four board‐certified obstetricians conducted this validation by reviewing 276 abstracted medical charts. They carried out this review in a blinded manner, without knowledge of the patients’ PE status.^2^ The diagnosis of PE was established following the guidelines outlined by the American Congress of Obstetricians and Gynecologists’ (ACOG).^29^ These guidelines include specific criteria for diagnosis, which encompass the presence of high blood pressure along with either proteinuria (≥300 mg per 24‐h collection or ≥ 1+ on urine dipstick) or the occurrence of elevated liver enzymes, a high platelet count, headache, or visual disturbances after reaching the 20th week of gestation. High blood pressure was defined as systolic blood pressure (BP) ≥ 140 mmHg, diastolic BP ≥ 90 mmHg, or both, with a second elevated measurement taken at least 4 h after the initial reading, as recorded in the medical record. The determination of total baseline 25OHD serum levels, conducted between the 10th and 18th weeks of gestation, was carried out using a chemiluminescence assay performed on the DiaSorin Liaison^®^ spectrometer at the DCC. This method, referred to as 25OHD TOTAL, has demonstrated a strong correlation with the DiaSorin 25‐hydroxyvitamin D RIA assay, which is considered the benchmark for establishing reference levels.^30^ The detection limit for the assay was ≤4.0 ng/mL. The coefficients of variation (CVs) for both inter‐assay and intra‐assay precision were found to be 11.2% and 8.1%, respectively. More comprehensive information regarding maternal 25OHD measurements and the quality control procedures applied, have been previously published.^27^, ^28^

### 2.4 Detection and quantification of circulating microRNAs

Total RNA, encompassing small RNA molecules, was extracted from 1 mL blood sample using the Norgen Biotek RNA isolation kit (Thorold, ON, Canada) following the manufacturer's prescribed protocol. RiboGreen measurements indicated an averaged concentration of 4 ng/μL of total RNA in a 20 μL sample. The RNA was quantified using the Nanodrop 8000 and checked for high integrity. The integrity of RNA samples was assessed using the Agilent 2100 Bioanalyzer, and purity of the samples was confirmed using the NanoDrop spectrophotometer and RNA Integrity Number (RIN) ≥8. The isolated RNA was then subjected to conversion into cDNA, with the resulting reverse‐transcriptase product subsequently preamplified using Megaplex PreAmp Primers and TaqMan PreAmp Master Mix, both of which are products from Applied Biosystems (Grand Island, NY). For quantitative PCR of microRNAs, TaqMan microRNA primers were procured from Life Technologies Megaplex RT Primers, Human Pool Set v3.0 (Omaha, NE). A total of 758 unique PCR assays per sample, encompassing 754 well‐characterised human miRNAs and 4 controls assays (RNU44, RNU48, U6, ath‐mir159), were arranged on the OpenArray plate. These assays were subsequently run on the QuantStudio 12K Flex Real‐Time PCR System, utilising the OpenArray Block (Life Technologies, Carlsbad, CA). MiRBase v21 was employed for the annotation of miRNAs, utilising symbol and accession number information.^31^ All data discussed in this study have been deposited in NCBI's Gene Expression Omnibus (GEO) and can be accessed through GEO Series accession number GSE85307 (http://www.ncbi.nlm.nih.gov/geo/query/acc.cgi?token = epajoakqppkvbub&acc = GSE85307).

### 2.5 MicroRNA quality control and expression profiling

**FIGURE 1:**
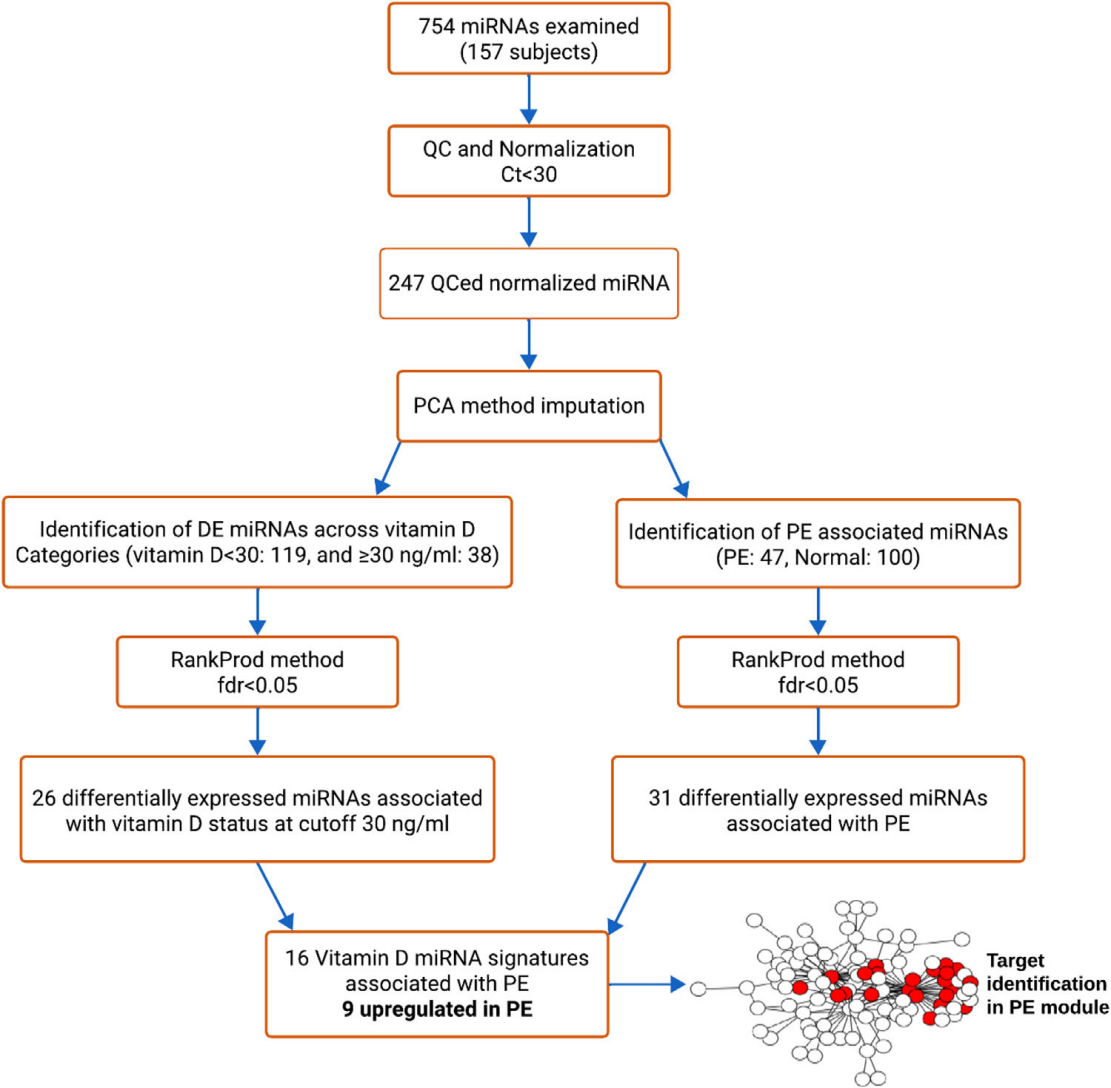
FIGURE 1 Flow chart of miRNA differential expression analysis. DE: differentially expressed, miRNA: microRNA, FDR: false‐discovery rate, PE: preeclampsia.

For quality assurance and per manufacturer protocol, only circulating miRNAs demonstrating good quality marks (i.e., amplification score < 1.1, Cq confidence value < 0.8) were considered (https://assets.thermofisher.com/TFS‐Assets/LSG/brochures/CO28730‐Crt‐Tech‐note_FLR.pdf). The quantile normalisation procedure for multisamples was applied to Ct values to normalise the distribution of expression.^32^ We set a threshold cycle value (*C_t_*) of < 30 as the cut‐off to define an abundance of miRNA; all miRNAs with an expression level ≥30 were excluded. To monitor potential haemolysis in the samples, we first ruled out any difference in expression abundance of hsa‐miR‐23a and hsa‐miR‐451 between participants who developed PE and those with a pregnancy with no adverse outcomes.^33^ Then, haemolysis was examined using ΔCq, calculated as the mean Cq of hsa‐miR‐23a minus the mean Cq of hsa‐miR‐451a. A ΔCq value greater than 5 was considered indicative of possible haemolysis, while a ΔCq value exceeding 7 conferred a high risk of haemolysis affecting the obtained data.^34^ All samples with a ΔCq value less than 5 included for further analysis. Additionally, any miRNA with more than 20% missing values indicating unmeasured expression among the participants was excluded from further analysis. Hence, in the differential miRNA expression analysis, a total of 231 out of 754 miRNAs (30.6%) were included. These miRNAs were derived from 157 samples collected during the 10−18 weeks of pregnancy, comprising 47 cases of PE and 110 women with normal pregnancies. Missing data points (nondetect values) were imputed using robust principal component analysis (PCA)^35^ as implemented in the Bioconductor package ‘pcaMethods’ and using the Bayesian PCA (bpca) method.^36^ This algorithm has shown tolerance to relatively high amounts of missing data (i.e., > 10%).^36^ Thereafter, we applied the same method used in our recent work for the investigation of PE‐mRNA signatures in the same cohort and their samples from the same time point.^2^ Hence, we performed surrogate variable analyses (SVA) and incorporated tests for differential miRNA expression while accounting for expression heterogeneity.^37^ When dealing with intricate datasets like gene and miRNA expressions, SVA offers the advantage of tackling multicollinearity among clinical predictors (e.g., BMI and vitamin D level) while also accounting for concealed sources of variation within expression data. In this process, we identified a total of eight surrogate variables (SVs). The expression data were subsequently adjusted in conjunction with the intervention arm to eliminate any batch‐related variations that were unrelated to our primary predictor of interest: the early‐pregnancy vitamin D status. Bioconductor package ‘RankProd’ was used for differential expression analysis on the Ct values, employing the rank product (RP) method to identify DE miRNAs.^38^ MiRNAs were ranked according to their significance in the rank product test statistic, as implemented by the RankProd R package. MiRNAs were considered significant when the false‐prediction percentage threshold was set to achieve a false‐discovery rate of less than .05.^39^ We independently identified circulating miRNA associated with two distinct factors in early pregnancy (10–18 weeks of gestation): baseline vitamin D status (classified as sufficient and insufficient at a cut‐off of 30 ng/mL) and the presence of PE. Subsequently, we determined the intersection of these two sets of DE miRNAs. The selection vitamin D cut‐off was guided by the collective findings from numerous studies that have explored the correlation between serum parathyroid hormone (PTH) and 25(OH)D levels in adults. These studies have consistently shown that the decline in PTH levels stabilised when the 25(OH)D level reaches around 30 ng/mL.^40^ It is important to emphasise that during pregnancy, serum PTH levels remain comparable to those seen in nonpregnant women.^41^, ^42^ Furthermore, we also used this vitamin D cut‐off to identify PE‐associated gene expressions in peripheral blood and the related gene module in our prior study.^2^ Figure 1 provides an overview of our approach. All analyses were conducted in R version 3.4 and 4.1, and Cystoscape version 3.9 was used for visualising, modelling, and analysing the targets of DE miRNAs in the PE module.^43^, ^44^

### 2.6 Preeclampsia module

#### 2.6.1 A PPI network of preeclampsia gene signatures

Previous studies have shown that a significant portion of the DE genes for a disease phenotype belong to a highly interconnected PPI network.^45^ Furthermore, biological networks of interacting genes could contain highly connected small and large components with interacting pathways, while preserving the function of the network as a whole.^46^ We extracted the seed genes of the PE gene signature, that is, those gene products that physically (directly) or indirectly (via one connector protein) interact with each other as mapped to the human PPI network. STRING database (STRINGdb v11), implemented in the Bioconductor package, was used to map the circulating PE gene signature (*N* = 348) to the human interactome network (PPI) and retrieve the PE disease module. In our previous work on the gene expression profiling of PE, we use the ‘HumanNet v.1’ PPI database (NCBI, March 2007) to map the PE gene signature, which covered only 16 243 protein‐coding genes.^2^ The recently updated STRING database (v11.5) significantly enhances its coverage of PPI data, encompassing a comprehensive collection of known and predicted protein interactions. This dataset comprises a total of 3 662 461 physical and functional annotations, involving 19 566 human proteins. These annotations are sourced from a variety of reputable databases, including BIND, DIP, GRID, HPRD, IntAct, MINT and PID for experimental data, as well as Biocarta, BioCyc, GO, KEGG and Reactome for curated data.^47^ Notably, the STRING database also includes predictions of functional PPIs, which encompass indirect associations between proteins. These predictions are derived from multiple sources, including gene cooccurrence in genomes (phylogenetic tree), gene coexpression, gene fusion events, genomic neighbourhood, text mining, and experimental data. These various sources of evidence are consolidated to generate a single score for each gene‐gene interaction, with scores ranging from 0 to 1, representing the strength of the predicted interaction. We used a score with at least medium confidence in prediction (> 0.4) that integrates the various available evidence on functional interaction between two proteins to map the PE gene signature. Accordingly, 563 274 biological interactions between 19 566 human proteins were included for construction of the PE module and subsequent integration of the module with PE‐associated miRNA signature (Table 2).

**TABLE 2:** Differentially expressed miRNAs associated with both vitamin D and preeclampsia status.

Differentially expressed miRNA	Regulation	Fold change	FDR
hsa‐miR‐885‐5p	Upregulated	1.48	.001
hsa‐miR‐122‐5p	Upregulated	1.46	.001
hsa‐miR‐34a‐3p	Upregulated	1.55	.001
hsa‐miR‐182‐5p	Upregulated	1.25	.001
hsa‐miR‐95‐3p	Upregulated	1.20	.003
hsa‐miR‐1244	Upregulated	1.18	.005
hsa‐miR‐545‐5p	Upregulated	1.18	.006
hsa‐miR‐642a‐5p	Upregulated	1.22	.017
hsa‐miR‐365a‐3p	Upregulated	1.26	.025
hsa‐miR‐29a‐5p	Downregulated	0.80	.002
hsa‐miR‐424‐5p	Downregulated	0.88	.008
hsa‐miR‐135a‐5p	Downregulated	0.77	.013
hsa‐miR‐378a‐5p	Downregulated	0.89	.013
hsa‐miR‐145‐3p	Downregulated	0.93	.014
hsa‐miR‐31‐5	Downregulated	0.88	.023
hsa‐miR‐144‐5p	Downregulated	0.93	.040

#### 2.6.2 Coherence of the PE transcriptome signature in the interactome (PPI)

We measured the PPI enrichment significance of the mapped PE gene signature (PE module) using the STRINGdb algorithm. The applied rule demonstrates whether a gene set of interest is significantly enriched in the PPI by comparing it to an expected number of edges for a random set of genes of a similar size selected from the genome. Furthermore, we used the Biological General Repository for Interaction Datasets (BioGRID 4.4: http://thebiogrid.org) as a secondary interaction database for estimating the PPI in humans to confirm independently the observed enrichment of the PE module in the STRINGdb interactome and whether interactions supported by the STRINGdb and the constructed module occupy a discrete disease neighbourhood.^48^ To this end, we measured the diameter of the disease module by calculating the average of the shortest paths’ lengths of all pairs of the module's nodes as follows:

$$\left\langle L\right\rangle =\frac{1}{N}\sum_{\begin{array}{c} s,t,\in \mathrm{Module}\\ s\neq t \end{array}}L^{s,t},$$

where

$$L_{s\neq t}^{s,t}=\mathrm{sp}\left(s,t,G\right)$$

and sp(s,t,G) measures the shortest path from node s to t on a graph (i.e., the network representing the connected disease module). To examine the significance of the closeness of the PE module elements as compared to random expectation (closeness of randomly selected proteins in the human interactome of the same size as the PE module), a z‐score was calculated as:

$$Z-\mathrm{score}\;=\;\frac{\langle L\rangle -\langle L^{\mathrm{rand}}\rangle }{\mathrm{std}\;\left(L^{\mathrm{rand}}\right)}.$$

The distribution of average shortest paths was obtained from 1000 randomly selected sets of genes in the PPI network of the same size as the PE module. A *z*‐score value less than −1.65 was considered significant.

### 2.7 Largest connected component of the PE module and peripheral components

After demonstration of the PE gene signature's residence in a discrete PPI neighbourhood, we divided the module into an observable PE module or the largest connected component (LCC) and a number of smaller components at the periphery of the LCC that were connected by indirect interactions (second order, i.e., > 2 edges) to the LCC. The LCC consisted of a maximal set of nodes such that each node was connected by a path demonstrating a direct interaction. The identified LCC and small satellite components were used to investigate the targets of differentially expressed PE‐related miRNAs (miRNA‐mRNA integration analysis). The topological characteristics of the LCC were also analysed and visualised and Gene Ontology (GO) pathways enrichment was conducted using g:Profiler (FDR < .05).^49^ Using the Bioconductor GOSemSim package,^50^ we estimated the overall similarity of Gene Ontology (GO) terms in three orthogonal ontologies – molecular function (MF), biological process (BP) and cellular component (CC) – for both the LCC and non‐LCC clusters. We also used the Human Protein Atlas (HPA v21.1; https://www.proteinatlas.org/) to pinpoint the subset of the PE module and LCC members exhibiting evidence of tissue‐specific expression in the placenta.

### 2.8 Identification of microRNA regulatory targets on the preeclampsia module

Several miRNA‐target databases have been developed to analyse and identify miRNA‐target interactions. Most of these databases are web‐based and contain manually curated experimentally validated miRNA‐gene interactions with in silico predicted targets determined through various algorithms. To enhance the precision of our target prediction and construct our miRNA‐gene regulatory network, we utilised the Bioconductor package miRNAtap. This tool enabled us to integrate ranked miRNA‐target predictions from various online sources and consolidate their prediction algorithm outputs, thereby enhancing the performance of each individual prediction and including experimentally derived targets. From the default sources in the miRNAtap databases,^51^ targets were aggregated from four of the most commonly cited prediction algorithm databases: DIANA,^52^ PicTar,^53^ TargetScan^54^ and miRDB.^55^ The union of all targets found, based on the geometric mean of the ranks, was taken as the set of targets for the DE miRNAs if they appeared in at least 2 of sources. Gene Ontology (GO) functional profiling of the products of mapped genes and LCC targets of the identified DE regulatory miRNAs was conducted using the g:Profiler package to examine whether they showed enrichment in any biological pathway independent of the connected PE module^47^ (FDR < .05). Evidence of tissue‐specific expression of targets in the placenta using the Human Protein Atlas (HPA, v21.1) was also explored.^56^

### 2.9 Validation of circulating microRNA signature expression in trophoblast cell lines

During the early stages of pregnancy, placenta is a dynamic and metabolically active organ, which is a major contributor of circulating miRNAs in the maternal bloodstream. Changes in miRNA expression in the placenta can reflect processes such as trophoblast invasion, angiogenesis and inflammation, which are closely related to pregnancy complications like hypertension.^57^ Hence, we first validated the expression of DE miRNAs in early‐pregnancy human trophoblast cells (HTR‐8/SVneo cell) from the physiologically relevant tissue of placenta.

#### 2.9.1 Cell culture

The HTR‐8/SVneo trophoblast cell line^58^ was obtained from American Type Culture Collection

(ATCC) and cultured in RPMI‐1640 with 5% foetal calf serum in a 5% CO_2_ incubator. Cells were passaged in T75 culture flasks and plated in 6‐well dishes for RNA isolation.

#### 2.9.2 Comparative microRNA analysis

Total RNA was isolated using the miRNeasy Mini kit (Qiagen) with the optional DNase I digestion step. The TaqMan^®^ MicroRNA Reverse Transcription Kit (ThermoFisher) was used for first strand synthesis and TaqMan^®^ MicroRNA assay kits were used for quantitative analysis of microRNAs. We utilised a PRISM 7900 HT Sequence Detector from Applied Biosystems to conduct real‐time TaqMan^®^Assays, employing the TaqMan^®^ Universal PCR Master Mix from Invitrogen. To determine the relative levels of the miRNAs, we employed the comparative ΔCt method,^59^ which involved normalising the data to *RNU48* gene expression. Each miRNA was measured in three separate experiments, with three biological replicas in each experiment, each assayed in triplicate. The probes assay kits used for miRNA detection are listed in Table S1.

#### 2.9.3 Cell transfection

The mirVana miRNA mimic for hsa‐miR‐182‐5p (ID MC12369) and negative control (NC) mimics were purchased from ThermoFisher. These miRNAs were transfected into cells using Lipofectamine 2000 (Thermo Fisher). Transfections of miRNA hsa‐miR‐182‐5p mimic were performed on plates of HTR‐8 SVneo cells at 50%−60% confluence, 24 h after plating. TaqMan probes were used to analyse hsa‐miR182‐5p/RNU 48 expression and relative IGF1R transcript levels 48 h posttransfection.

Western blot analysis was conducted to confirm decreased protein expression of IGF1R protein following transfection with the hsa‐miR‐182‐5p mimic. Antibodies to IGF1R beta (#9750) and GAPDH (#2118) for control were purchased from Cell Signaling Technology. Protein samples (8 μg) were loaded onto 4%−15% gradient SDS‐PAGE gels. After transfer of gels to PVDF membranes, blots were incubated with antibodies to IGF1R beta and GAPDH and then incubated with secondary antibodies linked to horse‐radish peroxidase. Bands were detected on X‐ray films through chemiluminescence imaging. The bands were captured using a ChemiDoc Touch imaging system (BioRad), and subsequent densitometry analysis was conducted using the Image Lab2 program (BioRad). To ensure accuracy, the band intensity of IGF1R was normalised by the intensity of the GAPDH band.

## 3 RESULTS

### 3.1 Differential expression of circulating microRNAs

Pregnant women who developed PE had 31 miRNAs that were differentially expressed relative to normal pregnancy controls (21/31 = 68% downregulated in PE, FDR < .05). Among the participants included in this study, 119 participants had insufficient vitamin D status (< 30 ng/mL, 41/47 = 87% with PE diagnosis) and 38 participants had sufficient (≥30 ng/mL, 6/47 = 13% with PE diagnosis). Twenty‐six miRNAs were identified as differentially expressed between women with 25OHD levels dichotomised at the 30 ng/mL cut‐off (7 downregulated in participants with insufficient vitamin D status [25OHD < 30 ng/mL], FDR < .05). The overlap between the miRNA signatures related to vitamin D and the miRNA signatures associated with PE delineated the vitamin D miRNA signatures linked to PE. Specifically, this intersection comprised 16 out of 31 (52%) miRNAs, of which 9 were upregulated under the conditions of PE. Supplemental File 2 provides the ranked list of DE miRNA associated with PE that were also associated with vitamin D (25OHD) status measured in peripheral blood (*N* = 16). The 8 top‐ranked up‐ and downregulated DE miRNAs [8/31] in pregnant women who developed PE as compared to those who continued a normal pregnancy were all previously reported in association with PE (upregulated: mir‐885, mir‐122, mir‐34a and mir‐182; downregulated: mir‐29a, mir‐27a, mir‐133a and mir‐424).^16^, ^60^, ^61^, ^62^, ^63^, ^64^, ^65^, ^66^ Seventy‐five per cent [12/16 = 75%] of PE‐vitamin D‐associated DE miRNAs had prior evidence in differential expression in circulating blood and/or placenta of pregnant women with PE as compared to those of normal pregnancies.^60^, ^61^, ^62^, ^63^, ^65^, ^67^, ^68^, ^69^, ^70^, ^71^, ^72^, ^73^, ^74^, ^75^, ^76^, ^77^, ^78^, ^79^, ^80^, ^81^, ^82^, ^83^, ^84^, ^85^, ^86^ Table S2 provides existing evidence in the literature for the association of each of these 16 DE miRNAs with PE in the literature. This observation was further substantiated by the evidence from microRNA Tissue Expression Database (miTED).^87^ According to miTED, hsa‐miR‐424‐5p and hsa‐miR‐182‐5p exhibited the highest levels of expression in the placenta tissue of pregnancies complicated with PE (Figure S3). Furthermore, hsa‐miR‐122‐5p and hsa‐miR‐182‐5p had the highest levels of expression in blood of pregnancies complicated with PE. Figure S4 demonstrates the expression of DE circulating miRNAs from this study across multiple tissues other than placenta in cumulative disease conditions in comparison to the healthy state using data from miTED.

### 3.2 Preeclampsia module

**FIGURE 2:**
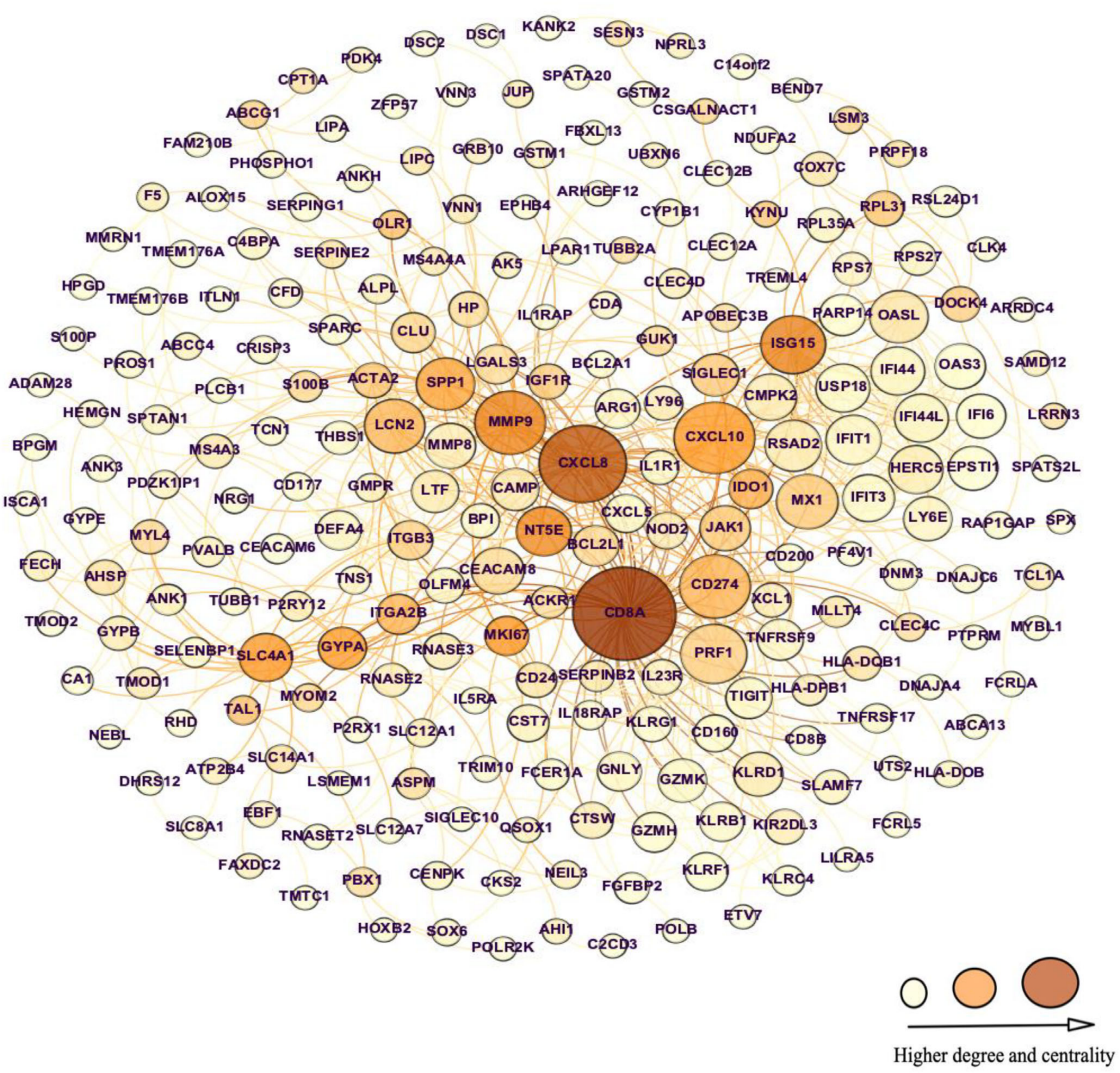
FIGURE 2 Observable preeclampsia (PE) module. This module was obtained from replication of the mRNA VDAART PE signature in an independent cohort (OMEGA) and mapped to the protein‐protein interaction network (PPI). The module consisted of the largest connected component (LCC) comprising gene products with evidence of direct (physical) interactions. The larger size of the nodes corresponds to higher connectivity (degree) and the darker colour of nodes corresponds to greater betweenness centrality.

In our previous report using HumanNetv1, we mapped 248 of the 348 (71%) replicated circulating genes associated with PE to the interactome. By using the most recent STRING PPI database (v11.5), the number of mapped genes was increased by 81 to comprise 329/348 (95%) genes (Figure 2 and Supplemental File 3). The related proteins of these signature genes demonstrated PPI enrichment, that is, having more interactions among themselves than expected for a random set of proteins of similar size (*N*_observed interaction_ = 5822 with average node degree of 5, *N*_expected interaction_ = 341; PPI enrichment *p* < .001). Using BioGrid as an independent PPI database, mapping of the PE gene signature also showed closeness of gene members and coherence in the PPI network measured by the average length of the shortest paths between the module members as compared with a random distribution of a gene set of the same size as the module members (*z*‐score = −5.47, Figure S1).

Of 329 mapped genes (PE module), 236 genes (71.73%) made up the LCC (Figure 2), and 93 genes made up the peripheral non‐LCC components (Figure S2 and Supplemental File 3). LCC and non‐LCC components demonstrated a GO sematic similarity measure of 0.87. Genes such as *CD8A*, *CXCL8 (IL8)*, *CXCL10*, *CD274*, *ISG15*, *PRF1*, *GYPA*, *MMP9, LCN2, CXCL5* and *NT5E* demonstrated the highest degree of connectivity (interaction) and betweenness centrality in the interactome (Supplemental File 6), all of which were previously reported to be associated with PE (Figure 2). Of PE module genes, 251 had evidence for expression in placental tissue (251/329 = 76.3%; *p for enrichment* < .0001; Supplemental File 3); 182 of these genes were members of the LCC (182/251 = 72.5%; *p for enrichment* < .0001), including all above‐mentioned genes with the highest degree of connectivity and betweenness centrality.

Functional profiling using GO annotation of the 236 members of the LCC components of the PE module showed enrichment of genes in several biological processes (GO: BP) and pathways highly ranked by immunologic functions (immune system process, *N* = 99; developmental process, *N* = 93; innate immune response, *N* = 51; inflammatory response, *N* = 35; response to cytokine, *N* = 35; individual FDR < .05) and cellular function (cell differentiation, *N* = 62; cell adhesion, *N* = 44; cell development, *N* = 43; individual FDR < .05; Supplemental File 4).

### 3.3 MicroRNA regulators of the preeclampsia module

**FIGURE 3:**
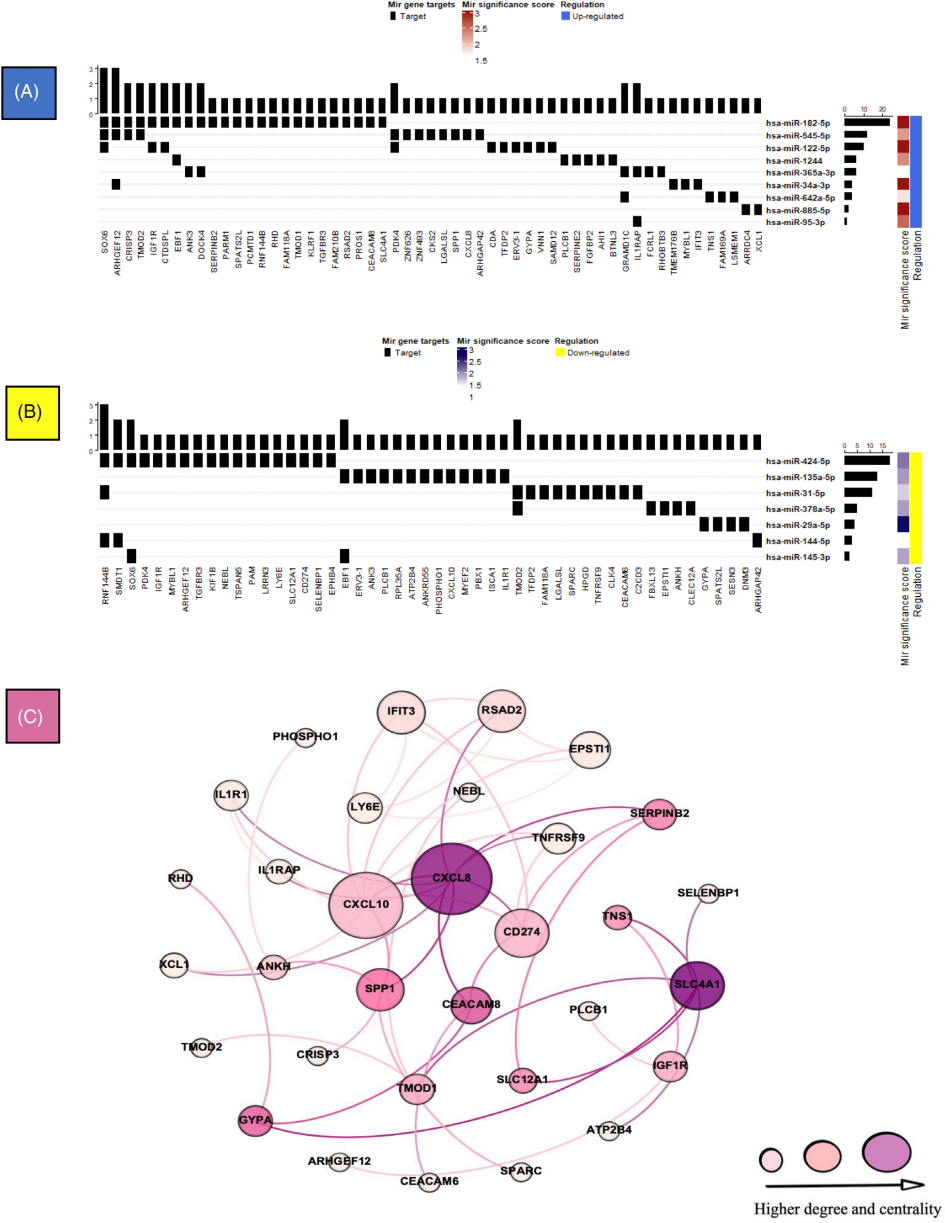
FIGURE 3 Differentially expressed (DE) miRNAs and their corresponding targets in the preeclampsia (PE) module. (**A)** Upregulated miRNAs in PE relative to normal pregnancy and their targets in the PE module. (**B)** Downregulated miRNAs in PE relative to normal pregnancy and their targets in the PE module. Significance score is log of the FDR value in differential expression analysis. **(C)** The connected subnetwork of miRNA targets in the LCC of the PE module. The larger size of the nodes corresponds to higher connectivity (degree) and the darker colour of nodes corresponds to greater betweenness centrality.

All 16 circulating DE miRNAs associated with both PE and vitamin D status were mapped to the four miRNA‐mRNA databases used for target identification and had targets in the PE module (both LCC and non‐LCC components) based on the agreement of their presence in at least two of the database interactions sources. Overall, these 16 DE miRNAs (9 upregulated) had 122 targets in the PE module (89 in the LCC), of which 87 were unique (64 in LCC, Supplemental File 3). Among 9 upregulated miRNAs, hsa‐miR‐182‐5p, hsa‐miR‐545 and hsa‐miR‐122 had the highest number of targets in the PE module (24, 12 and 10, respectively). Similarly, three miRNAs, hsa‐miR‐424‐5p, hsa‐miR‐135a‐5p and hsa‐miR‐31‐5p, had the greatest number of targets in the PE module among the downregulated miRNAs (18, 13, and 11, respectively; Figure 3A and B). Several upregulated and downregulated DE miRNAs had more than one target in the PE module and some DE mRNAs were targets of both upregulated and downregulated DE miRNAs (Figures 3A and B).

The network analysis of the DE miRNA targets demonstrated 32 targets comprising a connected subnetwork in the LCC with *CXCL8* and *CXCL10* having the highest degree and betweenness centrality (Figure 3C, 23 **downregulated**). Functional enrichment analysis of these 32 first degree connected targets showed immune response pathways, including regulation of Th1 and Th2 cytokine production, regulation of interleukin‐10 (IL‐10), cellular response to IL‐1, regulation of leukocyte migration, inflammatory response, and cell surface response signalling pathway (all FDR < .05, Supplemental File 5). Of note, 26 of these targets (26/32 = 81.3%) also had evidence of expression in the placenta.

### 3.4 Validating the expression of circulating microRNA signatures in the placenta

**FIGURE 4:**
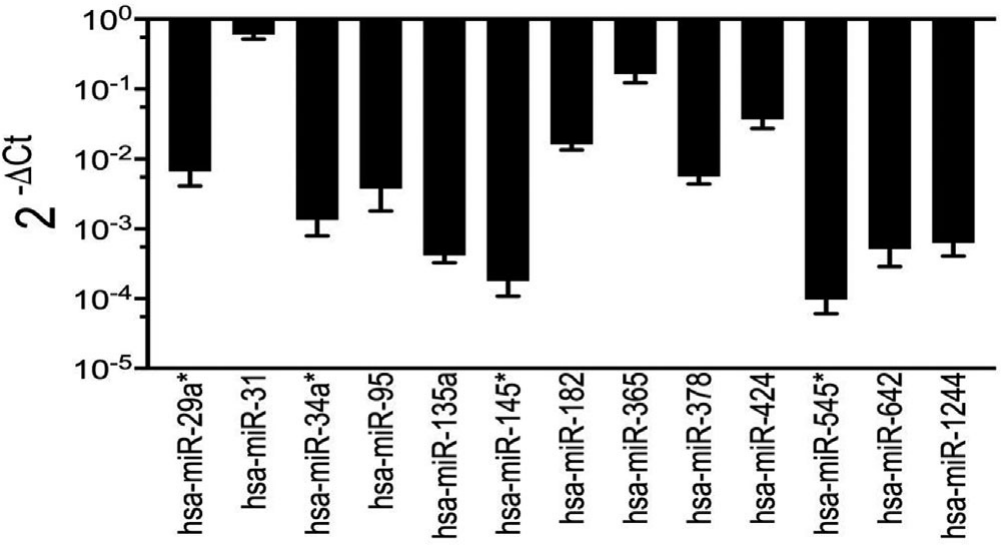
FIGURE 4 Relative expression levels of differentially expressed miRNAs following normalisation for the expression of endogenous control in HTR‐8/SVneo trophoblast cell line. Shown is the delta CT value relative to an endogenous control, *RNU48*. Thirteen miRNAs of the 16 (81.3%) differentially expressed miRNAs were detected in these cells.

Sixteen miRNAs that were found to be differentially expressed in blood samples and associated with vitamin D sufficiency were tested for their expression in the trophoblast cell line, HTR‐8/SVneo. Thirteen miRNAs with differential expression in peripheral blood were detectable in these cells; hsa‐miR‐855, hsa‐miR‐122 and hsa‐miR‐144 were not detected in this cell line. Figure 4 shows their relative levels of expression following normalisation with RNU48, an endogenous control.

**FIGURE 5:**
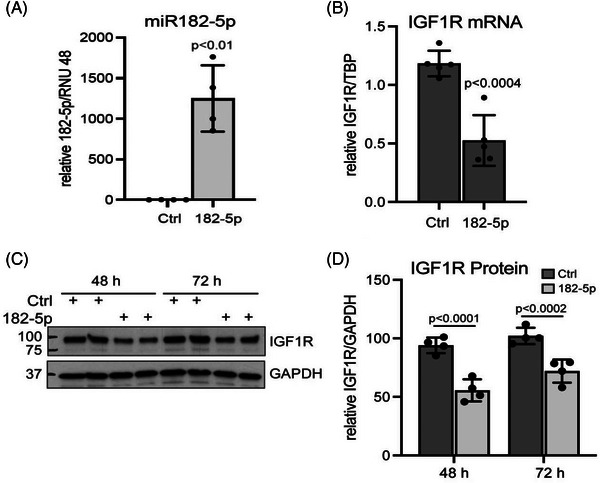
FIGURE 5 Cell Transfection of miR‐182‐5p and IGF1R expression. qRT‐PCR: Transfections of hsa‐miR‐182‐5p mimic (182‐5p) and control (Ctrl) were performed on plates of HTR‐8 SVneo cells at 50%−60% confluence, 24 h after plating. Samples were harvested 48 h post transfection for miRNA and IGFR1 RNA analysis or 48 and 72 h post transfection for protein analysis. (**A)** TaqMan probes were used to analyse hsa‐miR‐182‐5p and RNU48 expression (*n* = 4 independent experiments). Data were analysed by unpaired *t*‐test with Welch's correction for unequal variance. (**B)** TaqMan probes were used to analyse relative transcript levels of *IGF1R* and *TBP* by qRT‐PCR. Data were analysed by *t*‐test from 5 independent experiments. **(C, D)** Protein samples were used in a Western blot to monitor IGF1R beta and GAPDH expression 48 and 72 h post transfection (*n* = 4 independent experiments, with 2 technical replicas per experiment). Data were analysed by 2‐way ANOVA, followed by post hoc analysis. Note that the suppression of IGF1R with 182−5p is significantly different at 48 and 72 h (*p* < .05).

Figure S5 illustrates our approach for prioritising the miRNA signatures and their targets for in vitro experiments. IGF1 mediates its proliferative, antiapoptotic, trophoblast migration and invasion effects on the trophoblast through activating IGF1R.^88^ IGF1 also has a fundamental role in both prenatal and postnatal development and angiogenesis.^89^ *IGF1R* was a hub (higher degree and centrality) protein/gene in our PE module (Figure 2) as well as located in the LCC of targets of DE miRNAs (Figure 3C). It is also a putative target (downregulated) of hsa‐miR‐182‐5p (upregulated, Figure 3A). Hence, we aimed to validate the coexpression of miRNA‐182‐5p and *IGFR1* in HTR‐8/SVneo cells. After transfection of hsa‐miRNA‐182‐5p mimic, the relative abundance of miR182‐5p was confirmed using TaqMan probes after 48 h (*n* = 4, Figure 5A). Overexpression of hsa‐miR‐182‐5p mimic decreased IGF1R mRNA 48 h post transfection by 60% examined by qRT‐PCR (*n* = 5, Figure 5B). At the protein level (*n* = 4), the hsa‐miR‐182‐5p mimic decreased IGF1R protein (measured by the IGF1R beta chain) by 40% at 48 h and 30% at 72 h posttransfection compared to the expression in the presence of a negative control miRNA (Figures 5C and D).

## 4 DISCUSSION

Several studies have explored the association of circulating miRNAs with PE, but few of them have been conducted prior to the appearance of PE symptoms. Research investigating the implications of circulating miRNAs in early pregnancy and at the subclinical stage is currently limited.^90^ In this matched case‐control study, we first identified peripheral blood miRNAs that were DE in early pregnancy (10–18 weeks) in women who later developed PE compared to those with healthy pregnancies. Our prior results in the VDAART ITT population demonstrated that vitamin D sufficiency (25OHD ≥ 30 ng/mL) throughout pregnancy was associated with a decreased risk of PE development, as well as a dose‐response association in early pregnancy with respect to risk of PE development.^2^ We used this clinical feature in our case‐control genomic study to restrict the DE miRNAs to those associated with vitamin D sufficiency in early pregnancy. This approach allowed us to link a subset of our DE miRNAs with an orthogonal clinical and biological instrument associated with PE in the main study cohort to potentially decrease the false‐positive rate. In our previous work, we used the same study cohort and approach to investigate the DE circulating mRNAs from microarray data in early pregnancy of PE women, and replicated the identified gene signature in a second independent cohort.^2^ In this work, we mapped the DE gene signatures to a PPI network with high quality PPI interactions, constructed and visualised the PE transcriptome signature network, and explored the biological pathways of the PE disease module. We next integrated the DE miRNA signature with the mapped network of mRNA signature using aggregation of prior knowledge on the predicted miRNA‐mRNA interactions from multiple sources, and identified a potential regulatory subnetwork that includes key drivers of the PE module. The results demonstrate that the DE miRNAs target several gene products in the PE module, including some key genes with high degree of connectivity and betweenness centrality that have previously been associated with PE in placenta or peripheral blood. We further validated the expression of 13 of 16 (81%) DE miRNA signature in a trophoblastic cell line obtained from human placenta in early pregnancy.

### 4.1 PPI, preeclampsia module and biological processes

Protein‐protein interactions and those between proteins and other biomolecules provide a mechanistic basis for biological processes in which imbalances in physical association can alter the healthy state. The functional state of the network relies on the expression of protein nodes, which are governed by various regulatory mechanisms operating across different temporal and spatial dimensions.^91^ Endothelial dysfunction during angiogenesis, defective trophoblast differentiation and migration, and maternal‐foetal immune tolerance have been demonstrated to be key features of PE. The enrichment of replicated differentially expressed mRNA signatures in the PPI along with similarity of biological functions in the PE module (both LCC and non‐LCC components) indicate that the proteins encoded by the DE genes are biologically connected and occupy a discrete disease neighbourhood in the PPI. The enrichment of the PE gene signature for immunologic processes and cytokine production along with the observation of the association of a substantial number of these genes with PE in this and prior reports^2^ highlight the presence of an immune‐mediated condition and importance of the maternal‐foetal interface in early to mid‐stages of pregnancy and PE development. The enhanced systemic inflammatory response in women with PE is also emphasised by the observation of genes such as *CXCL8* (*IL‐8*), *CXCL10* and *CD8A*, as key regulators of the PE module, consistent with previous reports of dysregulation of these target genes in the disease.^92^, ^93^, ^94^, ^95^

These two CXCL genes, which directly interact with CD8A, were also part of the miRNA signature regulatory subnetwork in the LCC component of the PE module. The subnetwork enrichment in extracellular pathways (i.e., gene products that are secreted from a cell into interstitial fluid or blood) suggests placenta as a potential tissue source for further investigation. CXCL10 has potent antiangiogenic properties and promotes trophoblastic cell migration and invasion.^92^, ^96^ Chorionic tissue in the first trimester and placenta in the second trimester produces significant amount of CXCL8.^97^ CXCL8 plays a pivotal role in promoting trophoblast cell migration and invasion by increasing the expression levels of MMP‐2, MMP‐9, vascular endothelial growth factor (VEGF) and VEGF receptor. This cytokine also serves as a critical regulator of pathologic angiogenesis.^98^, ^99^, ^100^ Failure in these processes is tightly linked to the development of placental pathologies observed in PE.^101^

### 4.2 MicroRNA signatures and the preeclampsia module

MiRNAs, as posttranscriptional regulators of mRNA expression, could play an important role in the maintenance of the physiological status of the pregnant participants through gene networks, and, logically, their dysregulation might result in pathological processes. These pathological alterations have been demonstrated in both tissue and body fluids, including blood.^102^, ^103^ More specifically, miRNAs have recently received attention in PE due to their stability in the circulation and the ability of the placenta to release them into the circulation.^16^, ^17^, ^23^ These features and the fact that the placenta plays a critical role in the development and progression of PE suggests that additional analysis of the role of miRNAs in PE may lead to the identification of biomarkers for the subclinical stages of PE, as well as insights into the pathobiological mechanisms involved.^70^, ^104^, ^105^

#### 4.2.1 hsa‐miR‐424‐5p

The interaction between CD279 (PD‐1) and CD274 (PD‐L1) has been shown to be an important checkpoint in establishing maternal‐foetal tolerance and maintaining pregnancy, primarily by regulating T‐cell homeostasis.^106^ In one study, Toldi et al. reported lower expression of B7‐1 and B7‐2, but not B7‐H1 (CD274) proteins on peripheral monocytes in third trimester pregnancies of PE compared to normal pregnancies.^107^ Also, Mulliet et al. also showed that CD274 was downregulated in primary human trophoblasts by hypoxia, which is known to hinder placenta cell differentiation in PE.^108^ CD274 in our study was a key regulator of the PE module targeted by miRNA‐424 (Figure 3C). These observations along with prior evidence of the expression of B7‐H1 throughout pregnancy implicates the importance of PD‐1/PD‐L1 interaction during earlier stages of high‐risk pregnancies for PE and could be a target for trophoblast CD274‐mediated regulation during pregnancy.^109^, ^110^

#### 4.2.2 hsa‐miR‐378a‐5p

The enhancement effect of miRNA‐378 in spreading of extravillous trophoblast cells in first trimester placental explants has been previously demonstrated.^75^ This miRNA has also been detected in human placenta throughout pregnancy.^75^ This effect is postulated to be exerted through NODAL, a member of the transforming growth factor‐beta superfamily.^75^ miRNA‐378, consistent with a prior report,^75^ was downregulated among early pregnancy of women with PE in this study. We identified *NODAL* in the neighbourhood of the PE module and connected through *SERPINB2*, *SERPINE2* and *SERPING1* as well as *TGFBR3*, which suggests the functional similarity of several other genes in one pathway detected using ex vivo validation of a target in miRNA‐mRNA experimental studies.

#### 4.2.3 hsa‐miR‐34a‐3p

Inhibition of miR‐34a has been demonstrated to reverse miR‐34a‐induced apoptosis in the HTR‐8/SVneo human trophoblast cell line.^111^ This miRNA has also been upregulated in placentas from pregnancies with PE compared to normal placentas.^111^ Doridot et al. demonstrated overexpression of pri‐miR‐34a in PE placenta, particularly during the first trimester, although the mature miR‐34a was decreased in PE placenta.^62^ The investigators also showed that miR‐34a overexpression in JEG‐3 downregulates *SERPINA3*,^62^ a gene involved in trophoblast proliferation and invasion^112^ and its expression was found to be altered in placenta from pregnancies with PE.^113^ This gene is connected to the PE module through *SERPINB10*, *SERPINB2* and *SERPINE2*. However, we observed increased levels of circulatory miR‐34a‐3p in early pregnancy of PE women, suggesting a different expression profile of some miRNAs at preclinical and clinical stages of PE.

#### 4.2.4 hsa‐miR‐29a‐5p

A few studies have demonstrated the downregulation of mir‐29a in placenta and plasma of pregnancies with PE^23^, ^65^ but none have specifically described a role for miR‐29a‐5p, one of the miRNAs downregulated in our dataset. In a recent study, Zhou et al. showed the downregulation of mir29a‐3p in unpassaged (P0) human umbilical vein endothelial cells (HUVECs) isolated from caesarean‐section delivery, both from pregnancies characterised as normotensive and those with PE.^114^ Furthermore, knockdown of miR‐29a‐3p simultaneously with the similar miR‐29c‐3p in HUVECs was associated with inhibition of vascular endothelial growth factor A (VEGFA) and fibroblast growth factor 2(FGF2)‐induced endothelial function, which are key regulators of placental angiogenesis and vasodilation.^114^ *VEGFA* and *FGF2* are in the network neighbourhood of PE module in the PPI and connected to the module through *FGBFBP2*, *CXCL10*, and *CXCL8 (IL8)*.

#### 4.2.5 hsa‐miR‐182‐5p

Expression of hsa‐miR‐182‐5p has been observed to be elevated in placenta of pregnancies affected by PE compared to healthy pregnancies.^68^ Fang et al. corroborated these findings in placenta samples obtained from PE pregnancies. Their investigations revealed that the overexpression of hsa‐mir‐182‐5p inhibited the migratory capacity of HTR‐8/SVneo cells. Importantly, this inhibitory effect was reversed when hsa‐miR‐182‐5p was inhibited. Furthermore, the researchers demonstrated that simultaneous overexpression of RND3 and miR‐182‐5p partially counteracted the inhibitory effects of overexpressed miRNA‐182‐5p on migratory and invasive capacities of HTR‐8/SVneo cells.

RND3 is a small signalling G protein and appears to function as a negative regulator of cytoskeletal organisation, leading to a loss of adhesion. Consequently, it was suggested that RND3 plays a pivotal role in the pathobiology of PE associated with miR‐182‐5p.^84^ Notably, RND3 was situated within the neighbourhood of the subnetwork of DE miRNA targets within the LCC of PE module and in close proximity of CXCL8 and CXCL10. However, it is worth mentioning that in our previous gene expression analysis, RND3 mRNA did not exhibit differential expression in peripheral blood from participants with PE, nor was RND3 significantly affected by the overexpression of hsa‐miR‐182‐5p mimics in our in vitro studies (data not shown).

### 4.3 hsa‐miR‐182‐5p and IGF1R

In HTR‐8/SVneo cells, we observed that hsa‐miR‐182 had a downregulating effect on the expression of *IGF1R*. This relationship has also been demonstrated in peripheral blood mononuclear cells.^115^ Insulin‐like growth factors (IGFs) assume a critical role in orchestrating the distribution of placental resource to support foetal growth. This function extends throughout developmental stages and in response to environmental factors that impact the long‐term health of offspring. IGFs employ mechanisms that encompass the promotion of placental morphogenesis, facilitation of substrate transport and regulation of hormone secretion. These combined actions contribute to foetal growth, achieved either through the direct provision of essential nutrients and oxygen or indirectly by influencing maternal metabolic adaptation during pregnancy, thus ensuring the availability of nutrients for transplacental transport.^88^ Impaired trophoblast invasiveness and inadequate spiral arterial remodelling during early pregnancy are believed to contribute to poor placental perfusion, leading to foetal injury, growth restriction, and, in susceptible mothers, endothelial cell dysfunction, which collectively manifest as the clinical features of PE.^116^, ^117^, ^118^ IGF1 plays a multifaceted role in promoting placental health, as it inhibits apoptosis, stimulates proliferation and enhances the migration and invasion of trophoblast cells in both first trimester and term pregnancies.^88^ Furthermore, IGF1 also plays a role in promoting the differentiation of term trophoblast cells into syncytiotrophoblast. Additionally, it enhances the proliferation, invasion, and survival of first‐trimester human placental fibroblasts.^119^, ^120^, ^121^, ^122^ These proliferative and antiapoptotic effects of IGFs on trophoblasts are mediated through the activation of IGF1R, subsequently initiating the signalling pathways of MAPK and PI3K–AKT.^122^ Moreover, IGFs are known to induce trophoblast migration and invasion by acting through IGF1R and possibly INSR, followed by the activation of the MAPK and PI3K–AKT signalling pathways.^123^ Of note, IGF‐1 also stimulates the production of 1,25‐dihydroxyvitamin D [1,25‐(OH)_2_D] in the kidneys and placenta and is considered a crucial regulator of foetal growth. It is worth mentioning that lower maternal and umbilical cord serum levels of IGF‐I and 1,25‐(OH)_2_D have been observed in case of PE.^124^

## 5 LIMITATIONS

A significant constraint of this study lies in the utilisation of PCR arrays, which solely covered a limited number of miRNA probes, in contrast to the comprehensive catalogue of over 2000 known human miRNAs. Consequently, our investigation was confined to less than half of known miRNAs, leaving a substantial portion of miRNAs unexplored in our research. In addition, the small number of cases with adequate RNA did not allow us to explore the phenotype domains of PE; however, the majority of PE cases in the VDAART cohort and those included in the genomic study suffered from mild PE. Nevertheless, we used a nonparametric rank‐based method for differential expression of mRNAs and miRNAs; these methods make few or no assumptions about data structures in modelling differential expression of genes and thus run more robustly and outlier‐free. This approach enabled us to identify miRNAs and mRNAs that exhibited consistent upregulation or downregulation among participants with PE. Notably, none of the miRNAs in this study was previously reported to be associated with advancing gestational age between 4 and 16 weeks of pregnancy in terms of their circulating levels.^125^ Nevertheless, whether the miRNA signatures identified in this study also could show variability in expression during early pregnancy or be present later at clinical stage of PE or sourced from other tissues/organs affected by PE, and how these miRNAs could cause PE presentation and its severity is a matter of future research endeavours. Overall, miRNAs in the blood during the subclinical stage of pregnancy hypertension likely originate from a complex interplay of various tissues and cells, each contributing to the miRNA pool based on their specific roles in pregnancy and hypertension‐related processes. Research in this area is ongoing, aiming to identify specific miRNA signatures that could serve as early indicators of pregnancy hypertension and provide insights into the underlying molecular mechanisms.

Lastly, low 25(OH)D levels are associated with higher incidence or poorer outcomes for pregnancy complications including PE. In VDAART, we observed a dose‐response effect of vitamin D levels at 10−18 weeks of gestation in association with PE development.^2^ Hence, African Americans being prone to both lower levels of vitamin D and PE might face greater adverse effects related to vitamin D deficiency.^126^ Characterisation of race differences by vitamin D deficiency in association with PE and to what extent it might affect different RNA expressions in blood and tissues should be encouraged in future research endeavours.

## 6 CONCLUSIONS

This study profiles the expression of miRNAs in PE. We integrated two lines of omics data from the early pregnancy of matched case‐control pregnancies with and without PE. This systems biology and network medicine approach revealed several biological pathways and relevant miRNA‐mRNA signatures. These pathways and signatures denote potential biomarkers for early stage of PE development and suggest possible preventive measures requiring further exploration in functional and clinical experiments.

We have detected 16 DE circulating miRNAs in early pregnancy through a comparison of matched case‐control pregnancies with and without PE. Integrating these miRNAs with DE mRNA signatures associated with PE, employing systems and network biology approaches, has unveiled a range of biological pathways. Among these pathways, the IGF‐1 pathway stands out as potentially playing a pivotal role in the early pathophysiology of PE. Furthermore, the identification of these pathways and signatures has pointed towards potential biomarkers, such as hsa‐mir‐182‐5p, for the early stages of PE and has implications for the exploration of possible preventive measures.

## CONFLICT OF INTEREST STATEMENT

The authors declare no competing interests.

## Supporting information

Supporting information

Supporting information

Supporting information

Supporting information

Supporting information

Supporting information

## Data Availability

The data that support the findings of this study are openly available in NCBI Gene Expression Omnibus (GEO) database at https://www.ncbi.nlm.nih.gov/geo/query/acc.cgi?acc = GSE85307, reference number GSE85307.
