# Supplementary material for: Integration of circulating microRNAs and transcriptome signatures identifies early‐pregnancy biomarkers of preeclampsia
Source: Clin Transl Med. 2023 Oct 31;13(11):e1446. doi: 10.1002/ctm2.1446 (PMC10616748; doi:10.1002/ctm2.1446)

**Integration of Circulating microRNAs with Peripheral Blood Preeclampsia Transcriptome Signatures at Early Pregnancy Reveals Candidate Biomarkers**

Hooman Mirzakhani, MD, MMSc, PhD^1+*^; Diane Handy, PhD^2^; Zheng Lu, MS^1^; Ben Oppenheimer, MSc^1^; Augusto A. Litonjua, MD, MPH^3^; Joseph Loscalzo MD, PhD^2^; Scott T. Weiss, MD, MS^1^

^1^Channing Division of Network Medicine, Department of Medicine, Brigham and Women’s Hospital, Harvard Medical School, Boston, MA, USA

^2^Division of Cardiovascular Medicine, Department of Medicine, Brigham and Women's Hospital, Harvard Medical School, Boston, Massachusetts, USA

^3^Division of Pediatric Pulmonary Medicine, Department of Pediatrics, Golisano Children’s Hospital at Strong, University of Rochester Medical Center, Rochester, NY, USA

^+^Current address: Channing Division of Network Medicine, Brigham and Women’s Hospital, Boston, MA 02115, USA

^*^Corresponding author: Hooman Mirzakhani email: [hoomi@post.harvard.edu](mailto:hoomi@post.harvard.edu)

**Supplemental File 1 includes:**

**Table S1.** List of probes assay kits used for miRNA detection in trophoblast cell line.

**Figure S1.** Closeness (proximity) of preeclampsia (PE) module members in comparison to a random selected module in the interactome.

**Figure S2.** Preeclampsia module.

**Table S2.** Manual curation of differentially expressed miRNAs in association with PE in the literature.

**Figure S3.** Evidence on expression of DE circulating miRNA in PE using miRNA Tissue Expression Database (miTED).

**Figure S4.** Evidence on expression of DE circulating miRNA in placenta using miTED.

**Figure S5.** Flow chart of prioritizing the miRNA signature and its targets for in vitro experiments.

**Figure S6.** Clustered pairwise correlation matrix of differentially expressed miRNAs and mRNAs.

**TABLE S1.** List of probes assay kits used for miRNA detection in trophoblast cell line.

| **ITEM** | **Assay ID** | **Cat number** | **Company** |
| --- | --- | --- | --- |
| **hsa-miR-29a*** | # 002447 | 4427975 | ThermoFisher |
| **hsa-miR-31** | # 002279 | 4427975 | ThermoFisher |
| **hsa-miR-34a*** | # 002316 | 4427975 | ThermoFisher |
| **hsa-miR-95** | # 000433 | 4427975 | ThermoFisher |
| **hsa-miR-135a** | # 000460 | 4427975 | ThermoFisher |
| **hsa-miR-145*** | # 0002149 | 4427975 | ThermoFisher |
| **hsa-miR-182** | # 002334 | 4427975 | ThermoFisher |
| **hsa-miR-365** | # 001020 | 4427975 | ThermoFisher |
| **hsa-miR-378** | # 000567 | 4427975 | ThermoFisher |
| **hsa-miR-424** | # 000604 | 4427975 | ThermoFisher |
| **hsa-miR-545*** | # 002266 | 4427975 | ThermoFisher |
| **hsa-miR-642** | # 001592 | 4427975 | ThermoFisher |
| **hsa-miR-1244** | # 002791 | 4427975 | ThermoFisher |
| **hsa-miR-122** | # 002245 | 4427975 | ThermoFisher |
| **hsa-miR-144*** | # 002148 | 4427975 | ThermoFisher |
| **hsa-miR-885-5p** | # 002296 | 4427975 | ThermoFisher |


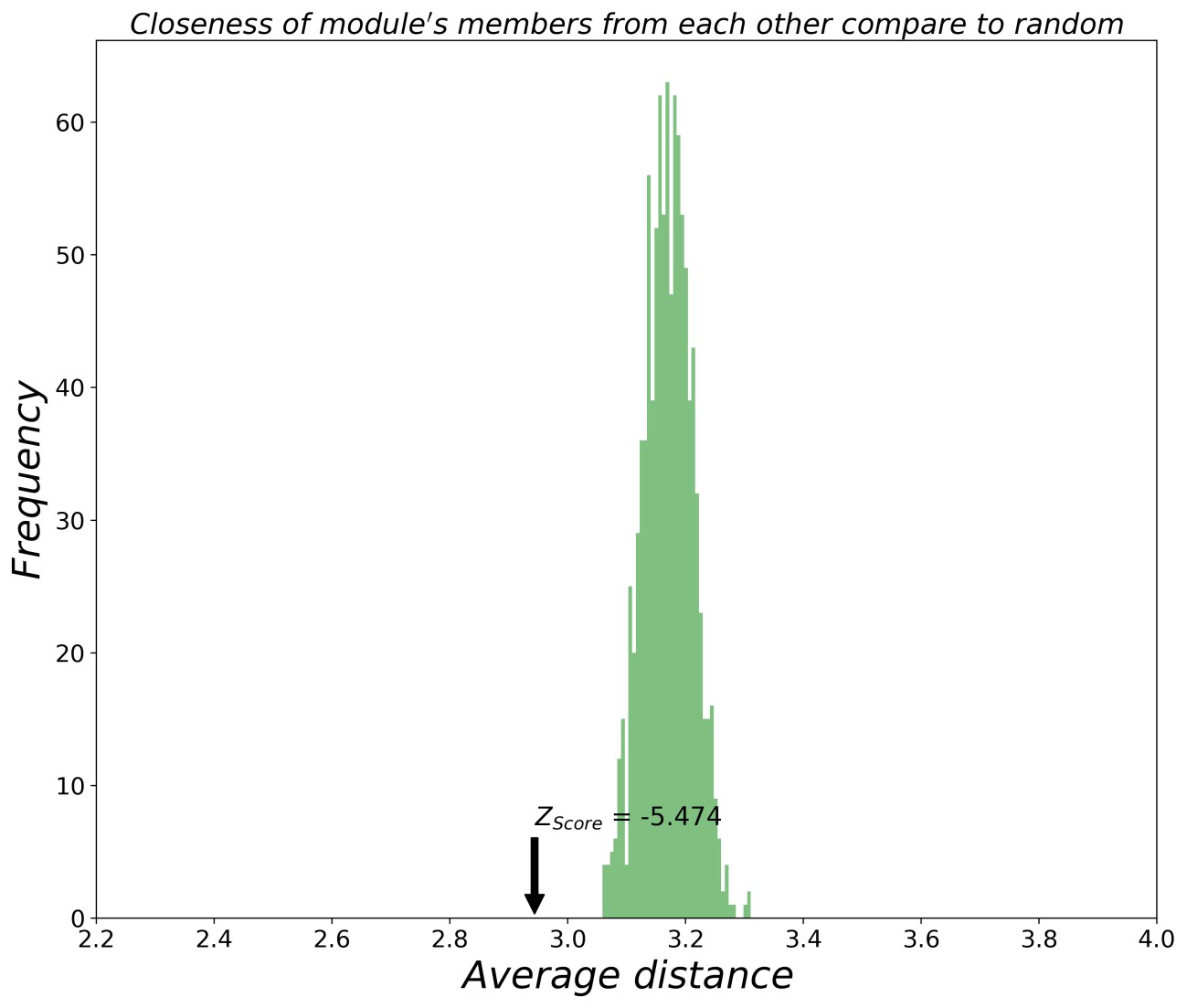


**Figure S1.** Closeness (proximity) of preeclampsia (PE) module members in comparison to a random selected module in the interactome using The Biological General Repository for Interaction Datasets (BioGRID).


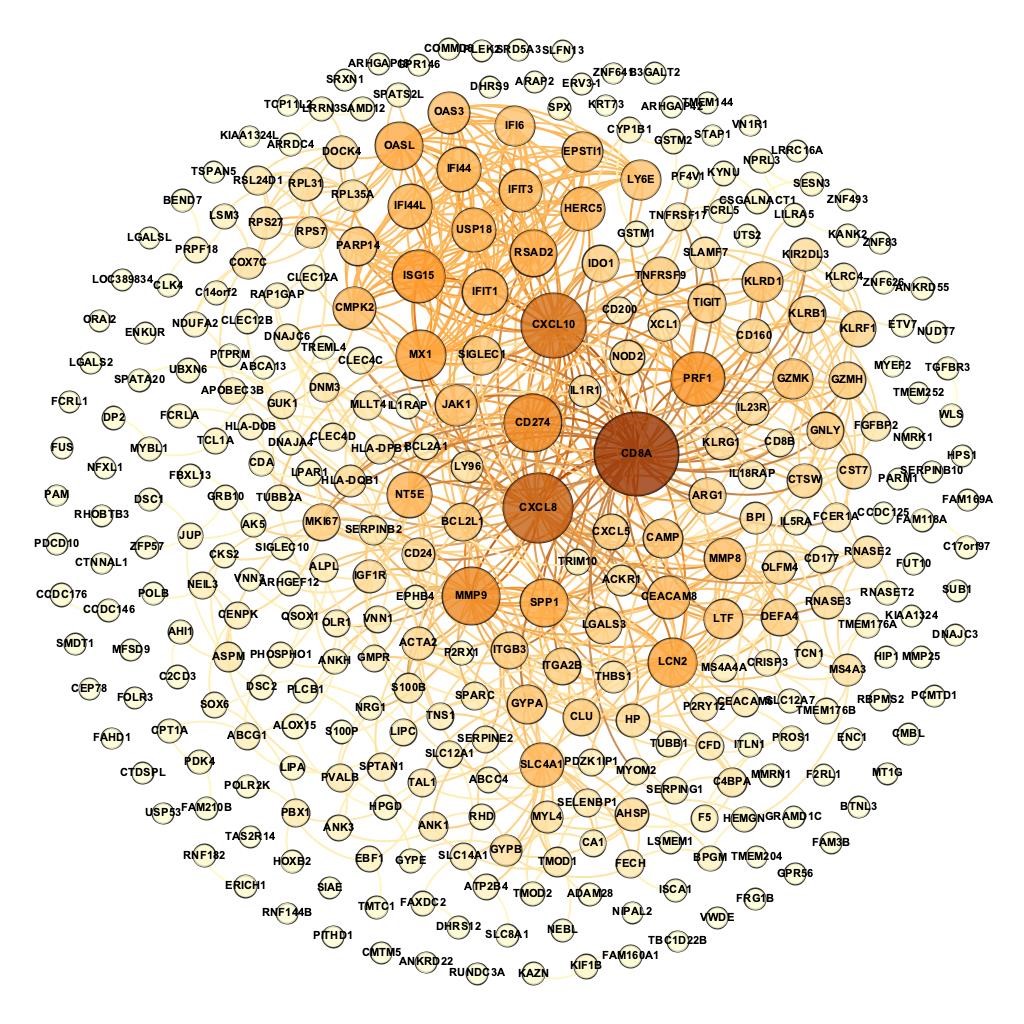


**Figure S2.** Preeclampsia module. This module was obtained from replication of the mRNA VDAART

PE signature in an independent cohort (OMEGA) and mapped to the protein-protein interaction network (PPI). The module consisted of the largest connected component (LCC) comprising gene products with evidence of direct (physical) interactions and a non-LCC component with non-direct (second order) interactions with the LCC. The larger size of the nodes corresponds to a higher connectivity degree and the darker color of nodes corresponds to greater betweenness centrality.

| **Table S2** Manual curation of differentially expressed miRNAs in association with PE in the literature | | | |
| --- | --- | --- | --- |
| Differentially Expressed | Directionality under the condition | Report in Association with Preeclampsia (PE) | Source |
| **hsa-miR-885-5p** | Up | Yes with replication in second cohort  -UP | Plasma ^60*^ |
| **hsa-miR-122-5p** | Up | Yes with validation by quantitative reverse transcriptase–polymerase chain reaction (qRT-PCR) in a second cohort for leukocytes but not placenta  -UP (both leukocytes and placenta) | Leukocytes ^67^ and placenta ^61^ |
| **hsa-miR-34a-3p** | Up (pri-miR-34a) | In PE-placental tissue only primary miRNA (pri-miR-34a) was up; the mature miR-34a was down in PE placentas. In JEG3 the time courses are complicated but pri-miR-34a was upregulated by hypoxia in JEG3 (24-36h) whereas mature miR-34a was initially decreased by hypoxia exposure (at-3-6h) | Placenta ^62^ |
| **hsa-miR-182-5p** | Up | Yes and miR-182*(3p) Yes  -Up validated using HTR-8/SVneov cell ^68,69^ | Placenta ^84^ |
| hsa-miR-95-3p | Up | No | - |
| hsa-miR-1244 | Up | No | - |
| hsa-miR-545-5p | Up | No | - |
| hsa-miR-642a-5p | Up | No | - |
| **hsa-miR-365a-3p** | Up | Yes | Serum ^70^ |
| **hsa-miR-29a-5p** | Down | Yes with validation  Up (Plasma and Placenta) ^63,65,71^ and Down (placenta) ^72^ also 29a-3p ^72^ Down | Plasma or serum  and Placenta ^63,65,71,72^ |
| **hsa-miR-424-5p** | Down | Yes  Down (severe PE) | Placenta ^73^ |
| **hsa-miR-135a-5p** | Down | Yes but 135b Up in placenta and serum ^74^  Down -Validated in HTR-8/SVneo cell | Placenta ^85^ |
| **hsa-miR-378a-5p** | Down | Yes  Down | Placenta ^75^ |
| **hsa-miR-145-3p** | Down | Yes, some with validation  Down ^76-78^ but 5p Up (trophoblast debris) ^78^ and down (placenta of early PE with IUGR) ^77^ | Placenta ^76-78^ |
| **hsa-miR-31-5p** | Down | Yes in exosomes extracted from the placenta and peripheral blood of pregnant women with preeclampsia and hsa-miR-31-3p down in Leukocytes ^67^ and Up in placenta with PE though not consistent with PCR ^79^ | Peripheral blood and placenta ^86^ |
| **hsa-miR-144-5p** | Down | Down  Reference 80 used HTR8/SVneo cells for validation; reference 81 used PCR for validation, reference 81 and 83 for severe PE | Placenta and serum from 12-14 weeks of gestation ^80-82^ |

* The numbers correspond to the references cited in the main text.

**Figure S3.** Examining the expression of differentially expressed circulating microRNAs in both placental tissue and plasma under preeclampsia status using microRNA Tissue Expression Database (miTED).


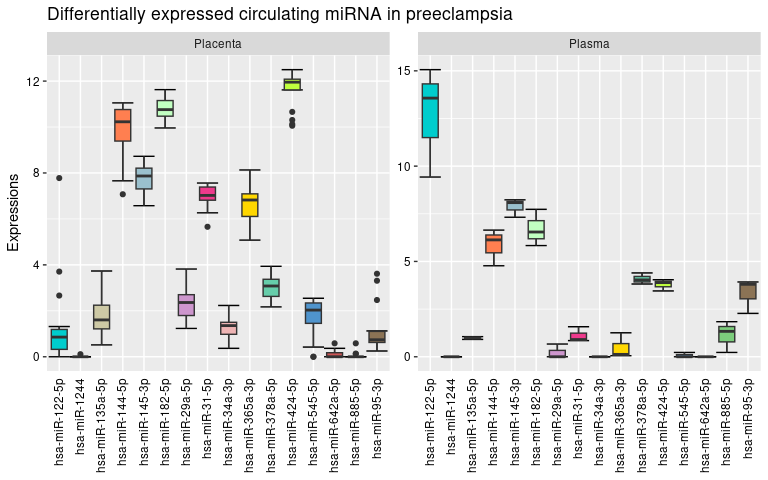


**Figure S4.** Examining the expression of differentially expressed circulating microRNA from this study in multiple tissues in disease conditions (across various diseases in comparison to the healthy state) using microRNA Tissue Expression Database (miTED).


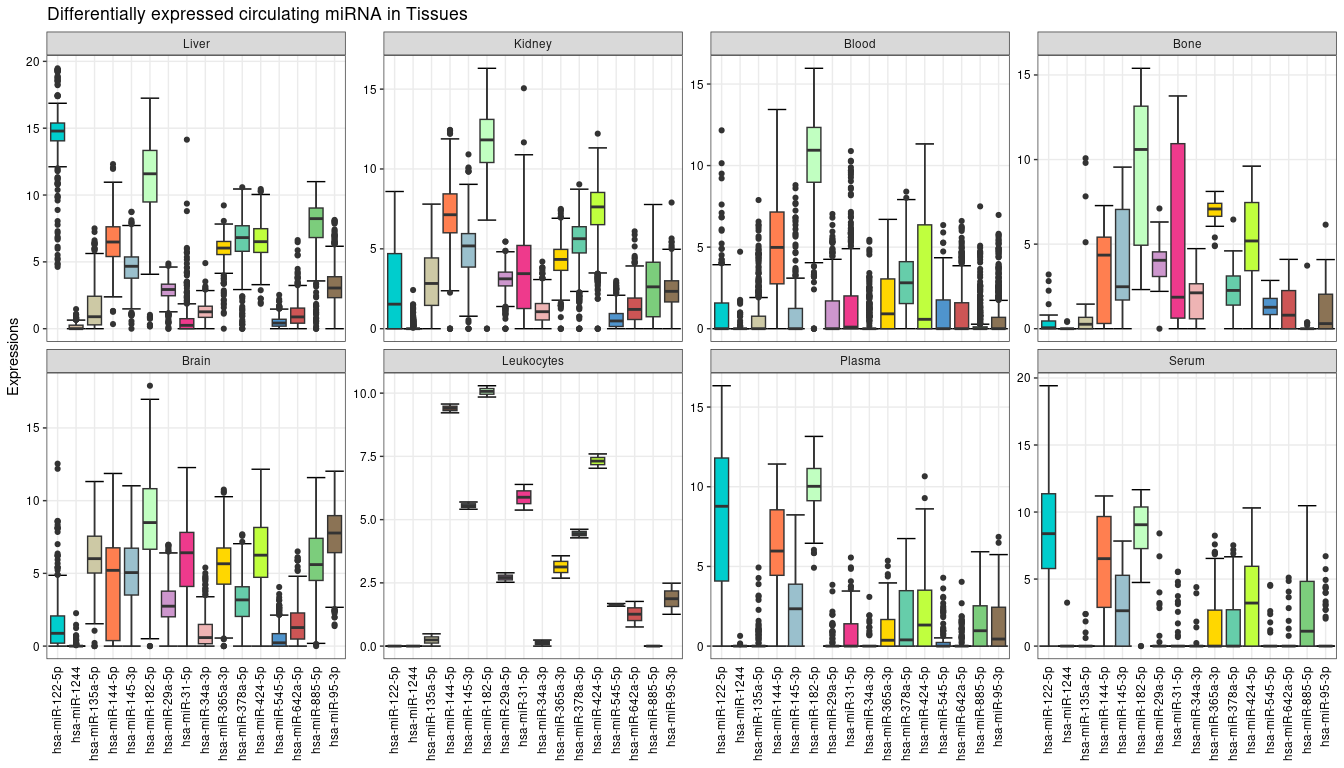


**Figure S5.** Flow chart of prioritizing the miRNA signature and its targets for in vitro experiments


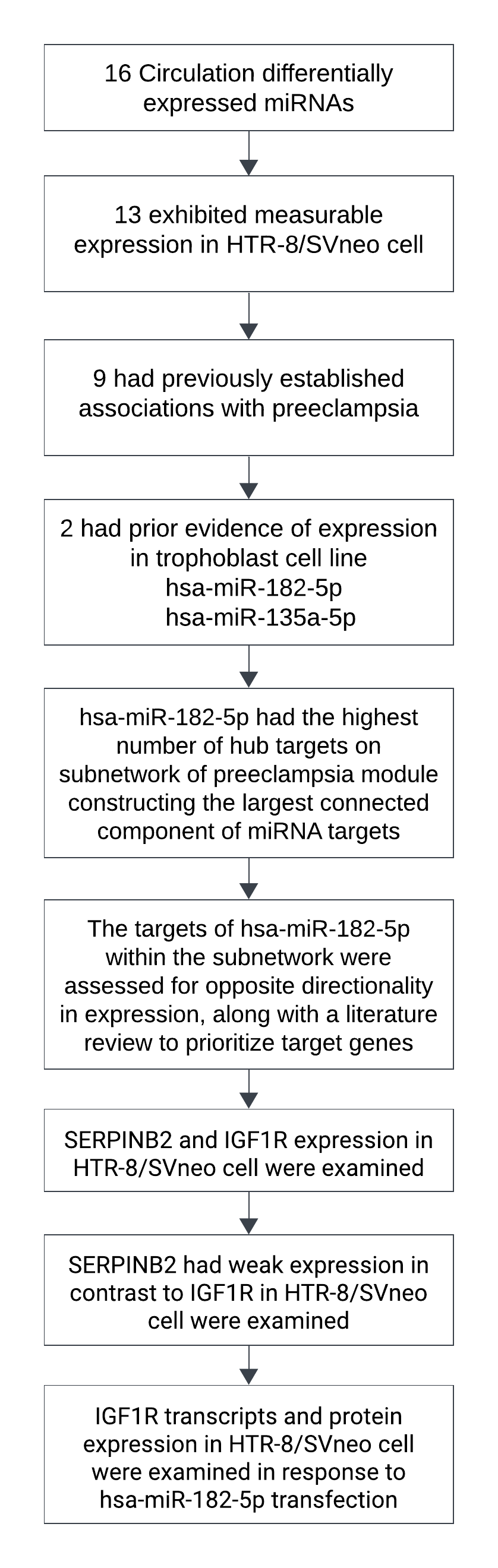


**Figure S6.** Clustered pairwise correlation matrix of differentially expressed miRNAs and mRNAs.


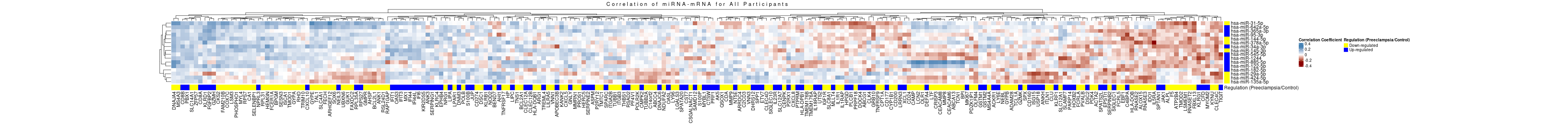

Supplement: Supplementary file 1 — Supporting information [file CTM2-13-e1446-s004.docx]
