# Supplementary material for: Integration of circulating microRNAs and transcriptome signatures identifies early‐pregnancy biomarkers of preeclampsia
Source: Clin Transl Med. 2023 Oct 31;13(11):e1446. doi: 10.1002/ctm2.1446 (PMC10616748; doi:10.1002/ctm2.1446)
Supplement: Supplementary file 2 — Supporting information [file CTM2-13-e1446-s002.docx]

**Integration of Circulating microRNAs with Peripheral Blood Preeclampsia Transcriptome Signatures at Early Pregnancy Reveals Candidate Biomarkers**

Hooman Mirzakhani, MD, MMSc, PhD^1+*^; Diane Handy, PhD^2^; Zheng Lu, MS^1^; Ben Oppenheimer, MSc^1^; Augusto A. Litonjua, MD, MPH^3^; Joseph Loscalzo MD, PhD^2^; Scott T. Weiss, MD, MS^1^

^1^Channing Division of Network Medicine, Department of Medicine, Brigham and Women’s Hospital, Harvard Medical School, Boston, MA, USA

^2^Division of Cardiovascular Medicine, Department of Medicine, Brigham and Women's Hospital, Harvard Medical School, Boston, Massachusetts, USA

^3^Division of Pediatric Pulmonary Medicine, Department of Pediatrics, Golisano Children’s Hospital at Strong, University of Rochester Medical Center, Rochester, NY, USA

^+^Current address: Channing Division of Network Medicine, Brigham and Women’s Hospital, Boston, MA 02115, USA

^*^Corresponding author: Hooman Mirzakhani email: [hoomi@post.harvard.edu](mailto:hoomi@post.harvard.edu)

**Supplemental File 2**

**Table I.** Differentially expressed miRNAs by preeclampsia (PE) status.

**Table II.** Differentially expressed miRNAs associated with vitamin D status.

**Table I.** Differentially expressed miRNAs by preeclampsia status.

| Differentially expressed miRNA | Regulations | RP.Rsum | Fold Change | FDR |
| --- | --- | --- | --- | --- |
| hsa-miR-885-5p | Upregulated | 38.54 | 1.48 | 0.001 |
| hsa-miR-122-5p | Upregulated | 42.20 | 1.46 | 0.001 |
| hsa-miR-34a-3p | Upregulated | 45.30 | 1.55 | 0.001 |
| hsa-miR-182-5p | Upregulated | 49.79 | 1.25 | 0.001 |
| hsa-miR-95-3p | Upregulated | 56.25 | 1.2 | 0.003 |
| hsa-miR-1244 | Upregulated | 58.47 | 1.18 | 0.005 |
| hsa-miR-545-5p | Upregulated | 59.15 | 1.18 | 0.006 |
| hsa-miR-642a-5p | Upregulated | 61.79 | 1.22 | 0.017 |
| hsa-miR-542-3p | Upregulated | 63.08 | 1.13 | 0.026 |
| hsa-miR-365a-3p | Upregulated | 63.32 | 1.26 | 0.025 |
| hsa-miR-29a-5p | Downregulated | 57.03 | 0.80 | 0.002 |
| hsa-miR-27a-5p | Downregulated | 59.31 | 0.80 | 0.004 |
| hsa-miR-133a-3p | Downregulated | 59.94 | 0.84 | 0.005 |
| hsa-miR-424-5p | Downregulated | 61.49 | 0.88 | 0.008 |
| hsa-miR-190a-5p | Downregulated | 61.76 | 0.78 | 0.008 |
| hsa-miR-517c-3p | Downregulated | 62.99 | 0.93 | 0.013 |
| hsa-miR-135a-5p | Downregulated | 63.42 | 0.77 | 0.013 |
| hsa-miR-378a-5p | Downregulated | 64.04 | 0.89 | 0.013 |
| hsa-miR-145-3p | Downregulated | 64.37 | 0.93 | 0.014 |
| hsa-miR-625-3p | Downregulated | 65.83 | 0.84 | 0.025 |
| hsa-miR-196b-5p | Downregulated | 65.87 | 0.82 | 0.023 |
| hsa-miR-31-5 | Downregulated | 66.02 | 0.88 | 0.023 |
| hsa-miR-422a | Downregulated | 66.59 | 0.88 | 0.027 |
| hsa-miR-212-3p | Downregulated | 67.34 | 0.78 | 0.034 |
| hsa-miR-512-3p | Downregulated | 67.87 | 0.89 | 0.039 |
| hsa-miR-886-3p | Downregulated | 67.92 | 0.89 | 0.038 |
| hsa-miR-517a-3p | Downregulated | 68.07 | 0.87 | 0.038 |
| hsa-miR-550a-5p | Downregulated | 68.11 | 0.85 | 0.036 |
| hsa-miR-369-5p | Downregulated | 68.21 | 0.95 | 0.036 |
| hsa-miR-616-5p | Downregulated | 68.60 | 0.86 | 0.039 |
| hsa-miR-144-5p | Downregulated | 68.83 | 0.93 | 0.040 |

**Table II.** Differentially expressed miRNAs associated with vitamin D status.

| **Differentially expressed miRNA** | **Regulation** | **RP.Rsum** | **Fold change** | **FDR** | **P.value** |
| --- | --- | --- | --- | --- | --- |
| **hsa-miR-182-5p** | Up | 44.00 | 1.39 | 0.010 | 0.001 |
| **hsa-miR-144-5p** | Up | 53.78 | 1.30 | 0.004 | < 0.001 |
| **hsa-miR-34a-3p** | Up | 59.32 | 1.13 | 0.030 | < 0.001 |
| **hsa-miR-29a-5p** | Up | 59.91 | 1.21 | 0.031 | < 0.001 |
| **hsa-miR-99a-5p** | Up | 60.21 | 1.31 | 0.029 | < 0.001 |
| **hsa-miR-148b-5p** | Up | 61.45 | 1.26 | 0.038 | 0.001 |
| **hsa-miR-135a-5p** | Up | 62.14 | 1.29 | 0.043 | 0.001 |
| **hsa-miR-885-5p** | Down | 42.57 | 0.73 | 0.010 | 0.001 |
| **hsa-miR-642a-5p** | Down | 54.59 | 0.75 | 0.003 | < 0.001 |
| **hsa-miR-122-5p** | Down | 54.89 | 0.79 | 0.002 | < 0.001 |
| **hsa-miR-145-3p** | Down | 56.78 | 0.84 | 0.004 | < 0.001 |
| **hsa-miR-338-3p** | Down | 58.83 | 0.81 | 0.005 | < 0.001 |
| **hsa-miR-545-5p** | Down | 59.21 | 0.86 | 0.005 | < 0.001 |
| **hsa-miR-204-5p** | Down | 60.19 | 0.79 | 0.007 | < 0.001 |
| **hsa-miR-224-5** | Down | 60.79 | 0.77 | 0.008 | < 0.001 |
| **hsa-miR-193a-5p** | Down | 60.90 | 0.77 | 0.007 | < 0.001 |
| **hsa-miR-424-5p** | Down | 61.73 | 0.96 | 0.009 | < 0.001 |
| **hsa-miR-95-3p** | Down | 63.07 | 0.87 | 0.014 | < 0.001 |
| **hsa-miR-483-5** | Down | 64.55 | 0.90 | 0.023 | 0.001 |
| **hsa-miR-654-3p** | Down | 65.01 | 0.90 | 0.026 | 0.001 |
| **hsa-miR-1244** | Down | 65.29 | 0.92 | 0.026 | 0.002 |
| **hsa-miR-378a-5p** | Down | 66.80 | 0.94 | 0.043 | 0.003 |
| **hsa-miR-625-5** | Down | 66.95 | 0.87 | 0.043 | 0.003 |
| **hsa-miR-31-5** | Down | 67.26 | 0.88 | 0.045 | 0.003 |
| **hsa-miR-143-3p** | Down | 67.34 | 0.81 | 0.044 | 0.003 |
| **hsa-miR-365a-3p** | Down | 67.38 | 0.86 | 0.042 | 0.003 |
