# Supplementary material for: Integration of circulating microRNAs and transcriptome signatures identifies early‐pregnancy biomarkers of preeclampsia
Source: Clin Transl Med. 2023 Oct 31;13(11):e1446. doi: 10.1002/ctm2.1446 (PMC10616748; doi:10.1002/ctm2.1446)
Supplement: Supplementary file 3 — Supporting information [file CTM2-13-e1446-s003.docx]

**Integration of Circulating microRNAs with Peripheral Blood Preeclampsia Transcriptome Signatures at Early Pregnancy Reveals Candidate Biomarkers**

Hooman Mirzakhani, MD, MMSc, PhD^1+*^; Diane Handy, PhD^2^; Zheng Lu, MS^1^; Ben Oppenheimer, MSc^1^; Augusto A. Litonjua, MD, MPH^3^; Joseph Loscalzo MD, PhD^2^; Scott T. Weiss, MD, MS^1^

^1^Channing Division of Network Medicine, Department of Medicine, Brigham and Women’s Hospital, Harvard Medical School, Boston, MA, USA

^2^Division of Cardiovascular Medicine, Department of Medicine, Brigham and Women's Hospital, Harvard Medical School, Boston, Massachusetts, USA

^3^Division of Pediatric Pulmonary Medicine, Department of Pediatrics, Golisano Children’s Hospital at Strong, University of Rochester Medical Center, Rochester, NY, USA

^+^Current address: Channing Division of Network Medicine, Brigham and Women’s Hospital, Boston, MA 02115, USA

^*^Corresponding author: Hooman Mirzakhani email: [hoomi@post.harvard.edu](mailto:hoomi@post.harvard.edu)

**Supplemental File 3**

PE module members and the status by LCC and non-LCC as well as target status and their expression in placenta.

| Gene name | mapped | LCC | Target of miRNA | Name of miRNA | Placenta expression |
| --- | --- | --- | --- | --- | --- |
| TMTC1 | 1 | 1 | 0 |  | TRUE |
| HLA-DQB1 | 1 | 1 | 0 |  | TRUE |
| ZFP57 | 1 | 1 | 0 |  | FALSE |
| TMEM176B | 1 | 1 | 1 | hsa-miR-34a-3p | TRUE |
| MMP25 | 1 | 0 | 0 |  | TRUE |
| BTNL3 | 1 | 0 | 1 | hsa-miR-1244 | FALSE |
| CLEC4C | 1 | 1 | 0 |  | TRUE |
| TMEM176A | 1 | 1 | 0 |  | TRUE |
| ARHGEF12 | 1 | 1 | 1 | hsa-miR-34a-3p, hsa-miR-182-5p, hsa-miR-545-5p, hsa-miR-424-5p | TRUE |
| FAM3B | 1 | 0 | 0 |  | TRUE |
| FAM118A | 1 | 0 | 1 | hsa-miR-182-5p, hsa-miR-31-5p | TRUE |
| NFXL1 | 1 | 0 | 0 |  | TRUE |
| OLFM4 | 1 | 1 | 0 |  | TRUE |
| TNFRSF17 | 1 | 1 | 0 |  | FALSE |
| MMP8 | 1 | 1 | 0 |  | TRUE |
| TRIM10 | 1 | 1 | 0 |  | FALSE |
| CLEC4D | 1 | 1 | 0 |  | TRUE |
| IL5RA | 1 | 1 | 0 |  | FALSE |
| LILRA3 | 0 | 0 | 0 |  | FALSE |
| ALOX15 | 1 | 1 | 0 |  | TRUE |
| POLR2K | 1 | 1 | 0 |  | FALSE |
| LOC389834 | 1 | 0 | 0 |  | FALSE |
| MS4A3 | 1 | 1 | 0 |  | TRUE |
| NEBL | 1 | 1 | 1 | hsa-miR-424-5p | TRUE |
| ARRDC4 | 1 | 1 | 1 | hsa-miR-885-5p | TRUE |
| TMOD2 | 1 | 1 | 1 | hsa-miR-182-5p, hsa-miR-545-5p, hsa-miR-378a-5p, hsa-miR-31-5p | TRUE |
| UBXN6 | 1 | 1 | 0 |  | TRUE |
| ENC1 | 1 | 0 | 0 |  | FALSE |
| NRG1 | 1 | 1 | 0 |  | TRUE |
| TFDP2 | 1 | 0 | 1 | hsa-miR-122-5p, hsa-miR-31-5p | TRUE |
| PVALB | 1 | 1 | 0 |  | TRUE |
| RSL24D1 | 1 | 1 | 0 |  | TRUE |
| RNASE3 | 1 | 1 | 0 |  | TRUE |
| PROS1 | 1 | 1 | 1 | hsa-miR-182-5p | TRUE |
| ABCC13 | 0 | 0 | 0 |  | FALSE |
| ITLN1 | 1 | 1 | 0 |  | TRUE |
| FCRL5 | 1 | 1 | 0 |  | FALSE |
| HEMGN | 1 | 1 | 0 |  | TRUE |
| PDZK1IP1 | 1 | 1 | 0 |  | TRUE |
| CSGALNACT1 | 1 | 1 | 0 |  | FALSE |
| CA1 | 1 | 1 | 0 |  | TRUE |
| TCL1A | 1 | 1 | 0 |  | TRUE |
| HTATSF1P2 | 0 | 0 | 0 |  | FALSE |
| OLR1 | 1 | 1 | 0 |  | FALSE |
| ITGB3 | 1 | 1 | 0 |  | TRUE |
| QSOX1 | 1 | 1 | 0 |  | FALSE |
| ENKUR | 1 | 0 | 0 |  | TRUE |
| IL1RAP | 1 | 1 | 1 | hsa-miR-95-3p, hsa-miR-365a-3p | TRUE |
| FUS | 1 | 0 | 0 |  | TRUE |
| ZNF641 | 1 | 0 | 0 |  | TRUE |
| ARG1 | 1 | 1 | 0 |  | TRUE |
| FECH | 1 | 1 | 0 |  | TRUE |
| TCP11L2 | 1 | 0 | 0 |  | TRUE |
| RPS7 | 1 | 1 | 0 |  | TRUE |
| TUBB1 | 1 | 1 | 0 |  | TRUE |
| TNFRSF9 | 1 | 1 | 1 | hsa-miR-31-5p | FALSE |
| SUB1 | 1 | 0 | 0 |  | TRUE |
| SLC8A1 | 1 | 1 | 0 |  | TRUE |
| S100P | 1 | 1 | 0 |  | TRUE |
| CENPK | 1 | 1 | 0 |  | FALSE |
| PCMTD1 | 1 | 0 | 1 | hsa-miR-182-5p | TRUE |
| CCDC125 | 1 | 0 | 0 |  | TRUE |

| Gene name | mapped | LCC | Target of miRNA | Name of miRNA | Placenta expression |
| --- | --- | --- | --- | --- | --- |
| KAZN | 1 | 0 | 0 |  | TRUE |
| BPI | 1 | 1 | 0 |  | TRUE |
| LSM3 | 1 | 1 | 0 |  | TRUE |
| AHSP | 1 | 1 | 0 |  | TRUE |
| CXCL8 | 1 | 1 | 1 | hsa-miR-545-5p | TRUE |
| CEACAM6 | 1 | 1 | 1 | hsa-miR-31-5p | TRUE |
| IGKC | 0 | 0 | 0 |  | FALSE |
| CMBL | 1 | 0 | 0 |  | TRUE |
| KANK2 | 1 | 1 | 0 |  | TRUE |
| RUNDC3A | 1 | 0 | 0 |  | TRUE |
| TUBB2A | 1 | 1 | 0 |  | TRUE |
| KIF1B | 1 | 0 | 1 | hsa-miR-424-5p | TRUE |
| COMMD6 | 1 | 0 | 0 |  | FALSE |
| VNN3 | 1 | 1 | 0 |  | FALSE |
| PDK4 | 1 | 1 | 1 | hsa-miR-122-5p, hsa-miR-545-5p, hsa-miR-424-5p | TRUE |
| IDO1 | 1 | 1 | 0 |  | TRUE |
| FCRLA | 1 | 1 | 0 |  | TRUE |
| EBF1 | 1 | 1 | 1 | hsa-miR-182-5p, hsa-miR-1244, hsa-miR-135a-5p, hsa-miR-145-3p | FALSE |
| MS4A4A | 1 | 1 | 0 |  | TRUE |
| TNS1 | 1 | 1 | 1 | hsa-miR-642a-5p | TRUE |
| LCN2 | 1 | 1 | 0 |  | TRUE |
| ANKRD55 | 1 | 0 | 1 | hsa-miR-135a-5p | TRUE |
| ABCA13 | 1 | 1 | 0 |  | FALSE |
| LPAR1 | 1 | 1 | 0 |  | FALSE |
| CD274 | 1 | 1 | 1 | hsa-miR-424-5p | TRUE |
| ARHGAP18 | 1 | 0 | 0 |  | TRUE |
| MYL4 | 1 | 1 | 0 |  | TRUE |
| NDUFA2 | 1 | 1 | 0 |  | TRUE |
| GYPE | 1 | 1 | 0 |  | FALSE |
| RBPMS2 | 1 | 0 | 0 |  | FALSE |
| CYP1B1 | 1 | 1 | 0 |  | TRUE |
| SLC14A1 | 1 | 1 | 0 |  | TRUE |
| DNAJA4 | 1 | 1 | 0 |  | TRUE |
| STAP1 | 1 | 0 | 0 |  | TRUE |
| GYPB | 1 | 1 | 0 |  | FALSE |
| LINC01270 | 0 | 0 | 0 |  | FALSE |
| HPS1 | 1 | 0 | 0 |  | TRUE |
| ANKRD22 | 1 | 0 | 0 |  | TRUE |
| RAP1GAP | 1 | 1 | 0 |  | TRUE |
| FAXDC2 | 1 | 1 | 0 |  | FALSE |
| FBXL13 | 1 | 1 | 1 | hsa-miR-378a-5p | TRUE |
| COX7C | 1 | 1 | 0 |  | TRUE |
| CRISP3 | 1 | 1 | 1 | hsa-miR-182-5p, hsa-miR-545-5p | TRUE |
| PARM1 | 1 | 0 | 1 | hsa-miR-182-5p | TRUE |
| CLU | 1 | 1 | 0 |  | TRUE |
| SPX | 1 | 1 | 0 |  | FALSE |
| LY96 | 1 | 1 | 0 |  | FALSE |
| RNASE2 | 1 | 1 | 0 |  | TRUE |
| RNASET2 | 1 | 1 | 0 |  | TRUE |
| CDA | 1 | 1 | 1 | hsa-miR-122-5p | TRUE |
| ATP2B4 | 1 | 1 | 1 | hsa-miR-135a-5p | TRUE |
| C14orf2 | 1 | 1 | 0 |  | FALSE |
| FCRL1 | 1 | 0 | 1 | hsa-miR-365a-3p | TRUE |
| SESN3 | 1 | 1 | 1 | hsa-miR-29a-5p | FALSE |
| ABCC4 | 1 | 1 | 0 |  | TRUE |
| VNN1 | 1 | 1 | 1 | hsa-miR-122-5p | TRUE |
| TCN1 | 1 | 1 | 0 |  | FALSE |
| DSC1 | 1 | 1 | 0 |  | TRUE |
| TMOD1 | 1 | 1 | 1 | hsa-miR-182-5p | TRUE |
| ERV3-1 | 1 | 0 | 1 | hsa-miR-122-5p, hsa-miR-135a-5p | TRUE |
| B3GALT2 | 1 | 0 | 0 |  | FALSE |
| CMTM5 | 1 | 0 | 0 |  | TRUE |

| Gene name | mapped | LCC | Target of miRNA | Name of miRNA | Placenta expression |
| --- | --- | --- | --- | --- | --- |
| TBC1D22B | 1 | 0 | 0 |  | FALSE |
| GMPR | 1 | 1 | 0 |  | TRUE |
| BCL2A1 | 1 | 1 | 0 |  | FALSE |
| MMRN1 | 1 | 1 | 0 |  | TRUE |
| PLEK2 | 1 | 0 | 0 |  | TRUE |
| RHD | 1 | 1 | 1 | hsa-miR-182-5p | TRUE |
| DNAJC3 | 1 | 0 | 0 |  | TRUE |
| SLC4A1 | 1 | 1 | 1 | hsa-miR-182-5p | TRUE |
| CTNNAL1 | 1 | 0 | 0 |  | FALSE |
| ADAM28 | 1 | 1 | 0 |  | TRUE |
| CAMP | 1 | 1 | 0 |  | TRUE |
| SELENBP1 | 1 | 1 | 1 | hsa-miR-424-5p | TRUE |
| BEND7 | 1 | 1 | 0 |  | TRUE |
| CPT1A | 1 | 1 | 0 |  | TRUE |
| P2RY12 | 1 | 1 | 0 |  | TRUE |
| BPGM | 1 | 1 | 0 |  | TRUE |
| PHOSPHO1 | 1 | 1 | 1 | hsa-miR-135a-5p | FALSE |
| CD200 | 1 | 1 | 0 |  | TRUE |
| LTF | 1 | 1 | 0 |  | TRUE |
| PF4V1 | 1 | 1 | 0 |  | TRUE |
| RPL31 | 1 | 1 | 0 |  | TRUE |
| LILRA5 | 1 | 1 | 0 |  | FALSE |
| F2RL1 | 1 | 0 | 0 |  | TRUE |
| HLA-DOB | 1 | 1 | 0 |  | TRUE |
| CXCL5 | 1 | 1 | 0 |  | TRUE |
| CTDSPL | 1 | 0 | 1 | hsa-miR-122-5p, hsa-miR-182-5p | TRUE |
| CKS2 | 1 | 1 | 1 | hsa-miR-545-5p | TRUE |
| FAHD1 | 1 | 0 | 0 |  | TRUE |
| PDCD10 | 1 | 0 | 0 |  | TRUE |
| HP | 1 | 1 | 0 |  | TRUE |
| PRPF18 | 1 | 1 | 0 |  | TRUE |
| FAM210B | 1 | 1 | 1 | hsa-miR-182-5p | TRUE |
| ALPL | 1 | 1 | 0 |  | TRUE |
| ISCA1 | 1 | 1 | 1 | hsa-miR-135a-5p | TRUE |
| ITGA2B | 1 | 1 | 0 |  | TRUE |
| SOX6 | 1 | 1 | 1 | hsa-miR-122-5p, hsa-miR-182-5p, hsa-miR-545-5p, hsa-miR-424-5p, hsa-miR-145-3p | TRUE |
| ORAI2 | 1 | 0 | 0 |  | TRUE |
| KIAA1324 | 1 | 0 | 0 |  | TRUE |
| GUK1 | 1 | 1 | 0 |  | TRUE |
| IGF1R | 1 | 1 | 1 | hsa-miR-122-5p, hsa-miR-182-5p, hsa-miR-424-5p | TRUE |
| F5 | 1 | 1 | 0 |  | FALSE |
| SERPINB2 | 1 | 1 | 1 | hsa-miR-182-5p | TRUE |
| GPR146 | 1 | 0 | 0 |  | FALSE |
| PITHD1 | 1 | 0 | 0 |  | TRUE |
| MMP9 | 1 | 1 | 0 |  | TRUE |
| TAL1 | 1 | 1 | 0 |  | TRUE |
| SLC12A1 | 1 | 1 | 1 | hsa-miR-424-5p | TRUE |
| LSMEM1 | 1 | 1 | 1 | hsa-miR-642a-5p | FALSE |
| TSPAN5 | 1 | 0 | 1 | hsa-miR-424-5p | FALSE |
| POLB | 1 | 1 | 0 |  | TRUE |
| GRAMD1C | 1 | 0 | 1 | hsa-miR-642a-5p, hsa-miR-365a-3p | TRUE |
| KYNU | 1 | 1 | 0 |  | TRUE |
| IL1R1 | 1 | 1 | 1 | hsa-miR-135a-5p | TRUE |
| LGALSL | 1 | 0 | 1 | hsa-miR-545-5p, hsa-miR-31-5p | TRUE |
| SIGLEC10 | 1 | 1 | 0 |  | TRUE |
| SERPINB10 | 1 | 0 | 0 |  | TRUE |
| LGALS3 | 1 | 1 | 0 |  | TRUE |
| TMEM144 | 1 | 0 | 0 |  | FALSE |
| GYPA | 1 | 1 | 1 | hsa-miR-122-5p, hsa-miR-29a-5p | TRUE |
| ACTA2 | 1 | 1 | 0 |  | TRUE |
| BCL2L1 | 1 | 1 | 0 |  | TRUE |
| FCER1A | 1 | 1 | 0 |  | TRUE |

| Gene name | mapped | LCC | Target of miRNA | Name of miRNA | Placenta expression |
| --- | --- | --- | --- | --- | --- |
| ANK1 | 1 | 1 | 0 |  | TRUE |
| C2CD3 | 1 | 1 | 1 | hsa-miR-31-5p | TRUE |
| CEACAM8 | 1 | 1 | 1 | hsa-miR-182-5p | TRUE |
| CD24 | 1 | 1 | 0 |  | TRUE |
| PAX8-AS1 | 0 | 0 | 0 |  | FALSE |
| C4BPA | 1 | 1 | 0 |  | TRUE |
| DEFA4 | 1 | 1 | 0 |  | TRUE |
| SPP1 | 1 | 1 | 1 | hsa-miR-545-5p | TRUE |
| APOBEC3B | 1 | 1 | 0 |  | FALSE |
| CXCL10 | 1 | 1 | 1 | hsa-miR-135a-5p | TRUE |
| ETV7 | 1 | 1 | 0 |  | FALSE |
| VWDE | 1 | 0 | 0 |  | FALSE |
| ERICH1 | 1 | 0 | 0 |  | TRUE |
| RNF182 | 1 | 0 | 0 |  | TRUE |
| CLEC12B | 1 | 1 | 0 |  | FALSE |
| CLEC12A | 1 | 1 | 1 | hsa-miR-378a-5p | TRUE |
| PTPRM | 1 | 1 | 0 |  | TRUE |
| CD177 | 1 | 1 | 0 |  | TRUE |
| S100B | 1 | 1 | 0 |  | TRUE |
| C17orf97 | 1 | 0 | 0 |  | TRUE |
| FOLR3 | 1 | 0 | 0 |  | FALSE |
| KRT73 | 1 | 0 | 0 |  | TRUE |
| UTS2 | 1 | 1 | 0 |  | FALSE |
| LINC00282 | 0 | 0 | 0 |  | FALSE |
| TREML4 | 1 | 1 | 0 |  | FALSE |
| LINC00189 | 0 | 0 | 0 |  | FALSE |
| USP53 | 1 | 0 | 0 |  | TRUE |
| IFI44 | 1 | 1 | 0 |  | TRUE |
| IFI44L | 1 | 1 | 0 |  | TRUE |
| SIGLEC1 | 1 | 1 | 0 |  | TRUE |
| TREML3P | 0 | 0 | 0 |  | FALSE |
| AK5 | 1 | 1 | 0 |  | TRUE |
| RSAD2 | 1 | 1 | 1 | hsa-miR-182-5p | TRUE |
| IFIT1 | 1 | 1 | 0 |  | TRUE |
| SERPING1 | 1 | 1 | 0 |  | TRUE |
| SLC12A7 | 1 | 1 | 0 |  | TRUE |
| NIPAL2 | 1 | 0 | 0 |  | TRUE |
| IFI6 | 1 | 1 | 0 |  | TRUE |
| OASL | 1 | 1 | 0 |  | TRUE |
| CFD | 1 | 1 | 0 |  | FALSE |
| USP18 | 1 | 1 | 0 |  | TRUE |
| SIAE | 1 | 0 | 0 |  | TRUE |
| MYOM2 | 1 | 1 | 0 |  | TRUE |
| SAMD12 | 1 | 1 | 1 | hsa-miR-122-5p | TRUE |
| THBS1 | 1 | 1 | 0 |  | TRUE |
| GSTM1 | 1 | 1 | 0 |  | TRUE |
| NT5E | 1 | 1 | 0 |  | TRUE |
| OAS3 | 1 | 1 | 0 |  | TRUE |
| HERC5 | 1 | 1 | 0 |  | TRUE |
| JUP | 1 | 1 | 0 |  | TRUE |
| LIPA | 1 | 1 | 0 |  | TRUE |
| SIGLEC16 | 0 | 0 | 0 |  | FALSE |
| PAM | 1 | 0 | 1 | hsa-miR-424-5p | TRUE |
| CTSW | 1 | 1 | 0 |  | TRUE |
| ZNF204P | 0 | 0 | 0 |  | FALSE |
| TAS2R14 | 1 | 0 | 0 |  | FALSE |
| ARHGAP42 | 1 | 0 | 1 | hsa-miR-545-5p, hsa-miR-144-5p | TRUE |
| ZNF83 | 1 | 0 | 0 |  | TRUE |
| ZNF493 | 1 | 0 | 1 | hsa-miR-545-5p | FALSE |
| KLRC4 | 1 | 1 | 0 |  | FALSE |
| MX1 | 1 | 1 | 0 |  | TRUE |
| LRRN3 | 1 | 1 | 1 | hsa-miR-424-5p | TRUE |
| GSTM2 | 1 | 1 | 0 |  | TRUE |

| Gene name | mapped | LCC | Target of miRNA | Name of miRNA | Placenta expression |
| --- | --- | --- | --- | --- | --- |
| HOXB2 | 1 | 1 | 0 |  | FALSE |
| MLLT4 | 1 | 1 | 0 |  | FALSE |
| GZMH | 1 | 1 | 0 |  | TRUE |
| NMRK1 | 1 | 0 | 0 |  | TRUE |
| DHRS12 | 1 | 1 | 0 |  | TRUE |
| DOCK4 | 1 | 1 | 1 | hsa-miR-182-5p, hsa-miR-365a-3p | FALSE |
| RPL35A | 1 | 1 | 1 | hsa-miR-135a-5p | FALSE |
| HLA-DPB1 | 1 | 1 | 0 |  | TRUE |
| IL23R | 1 | 1 | 0 |  | TRUE |
| MYEF2 | 1 | 0 | 1 | hsa-miR-135a-5p | TRUE |
| SMDT1 | 1 | 0 | 1 | hsa-miR-424-5p, hsa-miR-144-5p | TRUE |
| ARAP2 | 1 | 0 | 0 |  | TRUE |
| EPSTI1 | 1 | 1 | 1 | hsa-miR-378a-5p | TRUE |
| ACKR1 | 1 | 1 | 0 |  | TRUE |
| DSC2 | 1 | 1 | 0 |  | TRUE |
| ANK3 | 1 | 1 | 1 | hsa-miR-182-5p, hsa-miR-365a-3p, hsa-miR-135a-5p | TRUE |
| LGALS2 | 1 | 0 | 0 |  | TRUE |
| NPCDR1 | 0 | 0 | 0 |  | FALSE |
| SERPINB9P1 | 0 | 0 | 0 |  | FALSE |
| DNM3 | 1 | 1 | 1 | hsa-miR-29a-5p | FALSE |
| PBX1 | 1 | 1 | 1 | hsa-miR-135a-5p | TRUE |
| CD8A | 1 | 1 | 0 |  | TRUE |
| NOD2 | 1 | 1 | 0 |  | TRUE |
| KIAA1324L | 1 | 0 | 0 |  | FALSE |
| MFSD9 | 1 | 0 | 0 |  | TRUE |
| KIR2DS5 | 1 | 1 | 0 |  | FALSE |
| AHI1 | 1 | 1 | 1 | hsa-miR-1244 | TRUE |
| ISG15 | 1 | 1 | 0 |  | TRUE |
| TGFBR3 | 1 | 0 | 1 | hsa-miR-182-5p, hsa-miR-424-5p | TRUE |
| IL18RAP | 1 | 1 | 0 |  | TRUE |
| HPGD | 1 | 1 | 1 | hsa-miR-31-5p | TRUE |
| FRG1B | 1 | 0 | 0 |  | FALSE |
| SERPINE2 | 1 | 1 | 1 | hsa-miR-1244 | FALSE |
| JAK1 | 1 | 1 | 0 |  | TRUE |
| TMEM204 | 1 | 0 | 0 |  | TRUE |
| SPATA20 | 1 | 1 | 0 |  | TRUE |
| KLRF1 | 1 | 1 | 1 | hsa-miR-182-5p | FALSE |
| CMPK2 | 1 | 1 | 0 |  | TRUE |
| LIPC | 1 | 1 | 0 |  | FALSE |
| CCDC146 | 1 | 0 | 0 |  | TRUE |
| ASPM | 1 | 1 | 0 |  | FALSE |
| NPRL3 | 1 | 1 | 0 |  | TRUE |
| GZMK | 1 | 1 | 0 |  | TRUE |
| KLRG1 | 1 | 1 | 0 |  | TRUE |
| FAM160A1 | 1 | 0 | 0 |  | TRUE |
| SRXN1 | 1 | 0 | 0 |  | FALSE |
| TMEM252 | 1 | 0 | 0 |  | TRUE |
| MYBL1 | 1 | 1 | 1 | hsa-miR-34a-3p, hsa-miR-424-5p | TRUE |
| DNAJC6 | 1 | 1 | 0 |  | TRUE |
| CST7 | 1 | 1 | 0 |  | TRUE |
| TIGIT | 1 | 1 | 0 |  | FALSE |
| CD8B | 1 | 1 | 0 |  | TRUE |
| PRF1 | 1 | 1 | 0 |  | TRUE |
| PLCB1 | 1 | 1 | 1 | hsa-miR-1244, hsa-miR-135a-5p | TRUE |
| RPS27 | 1 | 1 | 0 |  | FALSE |
| GPR56 | 1 | 0 | 0 |  | FALSE |
| RNF144B | 1 | 0 | 1 | hsa-miR-182-5p, hsa-miR-424-5p, hsa-miR-31-5p, hsa-miR-144-5p | TRUE |
| EPHB4 | 1 | 1 | 1 | hsa-miR-424-5p | TRUE |
| ABCG1 | 1 | 1 | 0 |  | TRUE |
| FAM169A | 1 | 0 | 1 | hsa-miR-642a-5p | TRUE |
| LY6E | 1 | 1 | 1 | hsa-miR-424-5p | FALSE |
| XCL1 | 1 | 1 | 1 | hsa-miR-885-5p | TRUE |
| CCDC176 | 1 | 0 | 0 |  | FALSE |

| Gene name | mapped | LCC | Target of miRNA | Name of miRNA | Placenta expression |
| --- | --- | --- | --- | --- | --- |
| WLS | 1 | 0 | 0 |  | TRUE |
| GNLY | 1 | 1 | 0 |  | TRUE |
| KLRD1 | 1 | 1 | 0 |  | FALSE |
| FGFBP2 | 1 | 1 | 1 | hsa-miR-1244 | FALSE |
| SPARC | 1 | 1 | 1 | hsa-miR-31-5p | TRUE |
| SRD5A3 | 1 | 0 | 0 |  | TRUE |
| SPTAN1 | 1 | 1 | 0 |  | TRUE |
| SLAMF7 | 1 | 1 | 0 |  | TRUE |
| MKI67 | 1 | 1 | 0 |  | TRUE |
| GRB10 | 1 | 1 | 0 |  | TRUE |
| SMA4 | 0 | 0 | 0 |  | FALSE |
| FUT10 | 1 | 0 | 0 |  | TRUE |
| DHRS9 | 1 | 0 | 0 |  | TRUE |
| CD160 | 1 | 1 | 0 |  | FALSE |
| BMS1P20 | 0 | 0 | 0 |  | FALSE |
| NEIL3 | 1 | 1 | 0 |  | FALSE |
| NUDT7 | 1 | 0 | 0 |  | TRUE |
| VN1R1 | 1 | 0 | 0 |  | FALSE |
| TPTEP1 | 0 | 0 | 0 |  | FALSE |
| FLJ44896 | 0 | 0 | 0 |  | FALSE |
| CLK4 | 1 | 1 | 1 | hsa-miR-31-5p | TRUE |
| CEP78 | 1 | 0 | 0 |  | TRUE |
| RHOBTB3 | 1 | 0 | 1 | hsa-miR-365a-3p | FALSE |
| TARP | 0 | 0 | 0 |  | FALSE |
| NUDT16P1 | 0 | 0 | 0 |  | FALSE |
| KLRB1 | 1 | 1 | 0 |  | TRUE |
| ZNF626 | 1 | 0 | 1 | hsa-miR-545-5p | TRUE |
| ANKH | 1 | 1 | 1 | hsa-miR-378a-5p | FALSE |
| HIP1 | 1 | 0 | 0 |  | TRUE |
| SLFN13 | 1 | 0 | 0 |  | TRUE |
| P2RX1 | 1 | 1 | 0 |  | FALSE |
| PARP14 | 1 | 1 | 0 |  | TRUE |
| IFIT3 | 1 | 1 | 1 | hsa-miR-34a-3p | TRUE |
| MT1G | 1 | 0 | 0 |  | FALSE |
| LRRC16A | 1 | 0 | 0 |  | FALSE |
| SPATS2L | 1 | 1 | 1 | hsa-miR-182-5p, hsa-miR-29a-5p | TRUE |
