# Supplementary material for: Integration of circulating microRNAs and transcriptome signatures identifies early‐pregnancy biomarkers of preeclampsia
Source: Clin Transl Med. 2023 Oct 31;13(11):e1446. doi: 10.1002/ctm2.1446 (PMC10616748; doi:10.1002/ctm2.1446)

**Integration of Circulating microRNAs with Peripheral Blood Preeclampsia Transcriptome Signatures at Early Pregnancy Reveals Candidate Biomarkers**

Hooman Mirzakhani, MD, MMSc, PhD^1+*^; Diane Handy, PhD^2^; Zheng Lu, MS^1^; Ben Oppenheimer, MSc^1^; Augusto A. Litonjua, MD, MPH^3^; Joseph Loscalzo MD, PhD^2^; Scott T. Weiss, MD, MS^1^

^1^Channing Division of Network Medicine, Department of Medicine, Brigham and Women’s Hospital, Harvard Medical School, Boston, MA, USA

^2^Division of Cardiovascular Medicine, Department of Medicine, Brigham and Women's Hospital, Harvard Medical School, Boston, Massachusetts, USA

^3^Division of Pediatric Pulmonary Medicine, Department of Pediatrics, Golisano Children’s Hospital at Strong, University of Rochester Medical Center, Rochester, NY, USA

^+^Current address: Channing Division of Network Medicine, Brigham and Women’s Hospital, Boston, MA 02115, USA

^*^Corresponding author: Hooman Mirzakhani email: [hoomi@post.harvard.edu](mailto:hoomi@post.harvard.edu)

**Supplemental File 4:**

Gene Ontology (GO) enrichment analysis of preeclampsia module. The adjusted p-values correspond to the False Discovery Rate (FDR) values.

**A.** Molecular Function (MF).

**B.** Biological Process (BP).

**C.** Cellular Component (CC).

**A.**


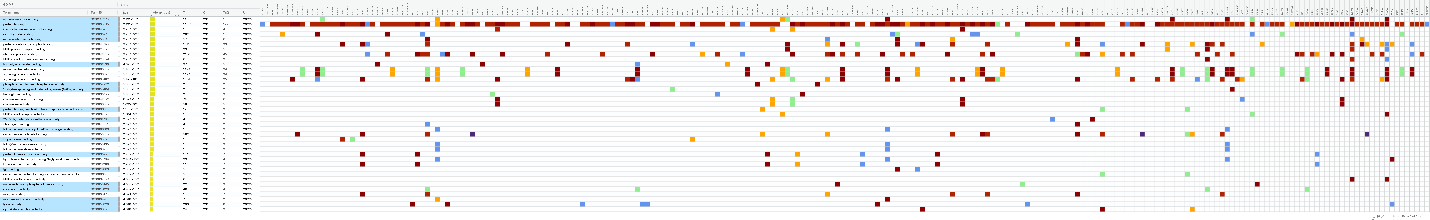


**B.**


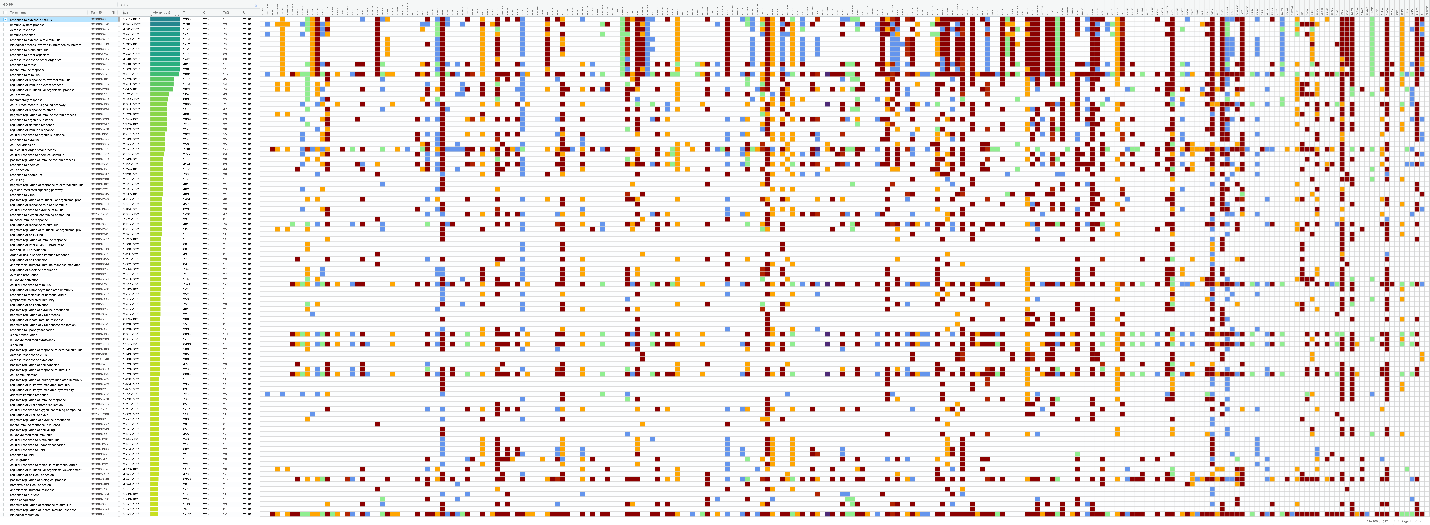


**C.**


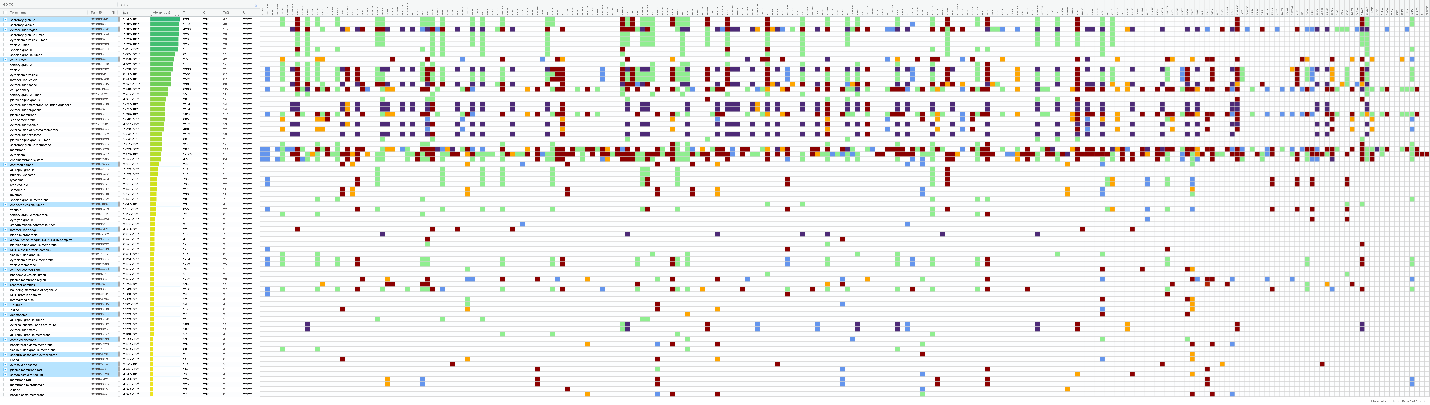

Supplement: Supplementary file 4 — Supporting information [file CTM2-13-e1446-s005.docx]
